# Age‐Dependent Regulation of Hippocampal Inflammation by the Mitochondrial Translocator Protein in Mice

**DOI:** 10.1111/acel.70039

**Published:** 2025-04-24

**Authors:** Kei Onn Lai, Jia Hui Wong, Nevin Tham, Lauren Fairley, Roshan Ratnakar Naik, Yulan Wang, Sarah R. Langley, Anna M. Barron

**Affiliations:** ^1^ Lee Kong Chian School of Medicine Nanyang Technological University Singapore Singapore Singapore; ^2^ Singapore Phenome Centre Nanyang Technological University Singapore Singapore; ^3^ School of Biosciences, Cardiff University Cardiff UK

**Keywords:** aging, hippocampus, LPS, mitochondria, neuroinflammation, translocator protein

## Abstract

The mitochondrial translocator protein (TSPO) is a biomarker of inflammation associated with neurodegenerative diseases, widely regarded to be upregulated in the aging brain. Here we investigated the interaction between aging and TSPO immunomodulatory function in the mouse hippocampus, a region severely affected in Alzheimer's Disease (AD). Surprisingly, we found that TSPO levels were decreased in brain innate immune populations in aging. Aging resulted in a reversal of TSPO knockout transcriptional signatures following inflammatory insult. TSPO deletion drastically exacerbated inflammatory transcriptional responses in the aging hippocampus, while dampening inflammation in the young hippocampus. This age‐dependent effect of TSPO was linked to NF‐kβ and interferon regulatory transcriptional networks. Drugs that disrupt the cell cycle and induce DNA damage, such as heat shock protein and topoisomerase inhibitors, were identified to mimic the inflammatory transcriptional signature characterizing aging in TSPO knockout mice most closely. These findings indicate that TSPO plays a protective role in brain aging. This TSPO–aging interaction is an important consideration in the interpretation of TSPO‐targeted biomarker and therapeutic studies, as well as in vitro studies that cannot model the aging brain.

## Graphical abstract

The role of mitochondrial translocator protein (TSPO) in hippocampal inflammation is age‐dependent, with TSPO deletion drastically exacerbating inflammation in aged mice, while dampening it in young mice. This TSPO–aging interaction was linked to NF‐kβ and interferon regulatory transcriptional networks. TSPO deletion exacerbated age‐dependent depletion of TCA cycle metabolite, fumarate, which is known to inhibit NF‐kβ activity. We propose that TSPO protects against hippocampal inflammaging via NF‐kβ to inhibit hallmarks of cellular senescence, including cell cycle disruption, DNA damage, and cell stress. Created in BioRender. https://BioRender.com/n20e027.

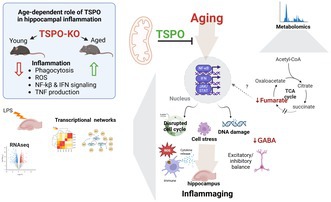

**Abbreviations**
AD: Alzheimer's disease
ADP: adenosine diphosphate
ATP: adenosine triphosphate
Cdk: cyclin‐dependent kinase
CMap: connectivity Map
CPM: counts per million
DEG: differentially expressed genes
DESeq2: differential gene expression analysis based on the negative binomial distribution
FDR: false discovery rate
FGSEA: fast gene set enrichment analysis
GABA: gamma‐aminobutyric acid
GI1: glioblastoma cell line
HOMER: hypergeometric optimization of motif enrichment
Hsp90: heat shock protein 90
Irf: interferon transcription factors
LINCS: Library of Integrated Network‐Based Cellular Signatures
Log2FC: log2 fold change
LPS: lipopolysaccharide
NAA: N‐acetylaspartate
NAD: nicotinamide adenine dinucleotide
NADH dehydrogenase: nicotinamide adenine dinucleotide hydrogen dehydrogenase
NAM: nicotinamide
NF‐kβ: nuclear factor kappa B
NMR: nuclear magnetic resonance spectroscopy
NPC: neural progenitor cell
PCSF: Prize‐collecting Steiner Forest
PERMANOVA: permutational multivariate analysis of variance
PLSDA: partial least squares determinant analysis
RIN: RNA integrity number
sscMap: statistically significant connections' map
STOCSY: statistical total correlation spectroscopy
STRING: Search tool for the retrieval of interacting genes/proteins
TCA cycle: tricarboxylic acid
TNF: tumor necrosis factor
TSPO: translocator protein
TSPO‐KO: translocator protein knockout
TSS: transcriptional start site
WGCNA: weighted gene co‐expression network analysis
WT: wild type

## 1 Introduction

Aging is tightly associated with chronic inflammation, often termed “inflammaging” (Li et al. 2023), both of which are important risk factors for cognitive decline, dementia, and delirium (Hou et al. 2019; Novoa et al. 2022). Inflammaging reflects age‐related changes in both peripheral and central immune function. As we age, immune senescence leads to increased susceptibility to infection, which has profound effects on the aging brain. Chronic inflammatory signals can impair memory and cognition and play an important role in the pathogenesis of neurodegenerative diseases, including Alzheimer's disease (AD). Key effectors of the inflammatory response in brain aging and disease are the resident innate immune cells of the brain, microglia, through a complex interplay of cell‐autonomous and non‐autonomous systemic‐brain interactions. These long‐lived cells are susceptible to dysfunction in age (Conde and Streit 2006; Hefendehl et al. 2014; Lopes et al. 2022; Olah et al. 2018; Thomas et al. 2022) and have been genetically implicated in AD pathogenesis (Kunkle et al. 2019; Raj et al. 2014). Aged microglia exhibit both impaired proliferative capacity and chronic overactivity, including increased proinflammatory cytokine release (Sierra et al. 2007; Ye and Johnson 1999), and dysfunctional immune defenses (Hefendehl et al. 2014; Stuesse et al. 2000; Thomas et al. 2022). For example, phagocytosis is impaired in aged microglia, which is an important protective function for mediating clearance of toxic beta amyloid that aggregates in the AD brain (Stuesse et al. 2000; Thomas et al. 2022). Additionally, aging is also characterized by systemic inflammation, resulting in increased circulating proinflammatory cytokines (Álvarez‐Rodríguez et al. 2012), leakiness of the blood–brain barrier (Hussain et al. 2021), with increased infiltration of inflammatory monocyte‐derived macrophages (Silvin et al. 2022). These inflammatory infiltrating macrophages have been observed clustered at sites of neuropathology in AD (Silvin et al. 2022), while other studies have associated markers of peripheral and brain inflammation with brain atrophy, cognitive decline, and AD risk (Cullen et al. 2021; Liang et al. 2024). Consequently, how aging and inflammation, also known as inflammaging, intersect to increase the risk of age‐associated cognitive decline and neurodegenerative disease is an important question.

The translocator protein, or TSPO, is a useful molecular imaging target to visualize brain inflammaging in health and disease (Bradburn et al. 2019; Cagnin et al. 2001; Dani et al. 2018; Edison et al. 2008; Fan et al. 2015; Hamelin et al. 2016; Kreisl et al. 2013; Parbo et al. 2017; Schaum et al. 2020; Varrone et al. 2015; Versijpt et al. 2003; Yasuno et al. 2012; Yokokura et al. 2017). Using positron emission tomography, brain TSPO signals have been found to significantly correlate with age, AD pathology, and cognitive deficits (Dani et al. 2018; Edison et al. 2008; Fan et al. 2015; Finze et al. 2023; Hamelin et al. 2016; Kreisl et al. 2013; Parbo et al. 2017; Pascoal et al. 2021; Versijpt et al. 2003). Although ubiquitously expressed, TSPO expression is upregulated in microglia and macrophages in response to inflammation (MacAskill et al. 2021; Nutma et al. 2021). For example, TSPO‐PET has been used to image microglial inflammation in the human brain in response to systemic LPS administration (Sandiego et al. 2015).

A mitochondrial protein, TSPO is functionally important in microglial metabolic and immune responses. We and others have shown that genetic deletion of TSPO impairs microglial bioenergetics (Milenkovic et al. 2019) and phagocytosis, worsening pathogenesis in models of AD (Fairley et al. 2023; Pradhan et al. 2023; Zhang, Wang, et al. 2021). Beyond its role in inflammation, TSPO has also been functionally implicated in aging, with a recent genetic study in drosophila showing that glial TSPO enhances longevity (Jullian et al. 2024). Functionally, TSPO has been implicated in the control of cellular bioenergetics (Hirsch et al. 1989; Liu et al. 2017), reactive oxygen species production (Guilarte et al. 2016; Loth et al. 2020), mitochondrial calcium homeostasis (Gatliff et al. 2017), mitochondrial cholesterol flux (Taylor et al. 2014) and mitochondrial–nuclear signaling (Desai et al. 2020). At the molecular level, TSPO interacts with key metabolic and inflammatory pathways in microglia and macrophages, NADPH oxidase, a regulator of redox balance (Guilarte et al. 2016; Loth et al. 2020) and NF‐Kβ signaling, a major inflammatory pathway (Horiguchi et al. 2019; Zhao et al. 2011). TSPO‐dependent regulation of redox via NADPH oxidase has been linked to mitochondrial calcium homeostasis (Gatliff et al. 2017), while TSPO‐mediated mitochondrial cholesterol flux has long been thought to play a role in steroidogenesis, a function supported by recent genetic studies in microglia and AD models (Bader et al. 2019; Pradhan et al. 2023). Interestingly, TSPO‐mediated cholesterol flux has also been linked to NF‐κB activation via retrograde mitochondrial–nuclear signaling (Desai et al. 2020). Meanwhile, other studies have found NF‐κB to be an upstream regulator of TSPO expression in microglia (Da Pozzo et al. 2019). Together, these findings highlight TSPO as a critical regulator of mitochondrial homeostasis, redox balance, and immune function, linking metabolic processes to inflammatory signaling and neurodegenerative disease processes.

Given mitochondrial and metabolic dysfunction is a universal hallmark of aging (López‐Otín et al. 2013), and increasing evidence indicates that mitochondrial metabolism plays a key role in coordinating immune processes (Fairley et al. 2021), TSPO may provide an interface between aging and inflammation at the mitochondria in brain innate immune cells. Here we investigated the potential interaction between TSPO immune function and aging in the mouse hippocampus, a region critically involved in memory and highly vulnerable to both AD and age‐related cognitive decline. We used a systemic LPS inflammatory stimulus to examine the role of aging in immune senescence, as LPS is well‐established model for age‐ and disease‐associated inflammation, mimicking both neuropathological and cognitive changes observed in age, dementia, and delirium (Ang et al. 2023; Bahaidrah et al. 2022; Brown and Heneka 2024; Kealy et al. 2020; Nishiguchi et al. 2024; Sultan et al. 2021; Zhang, Ma, et al. 2021).

## 2 Materials and Methods

### 2.1 Animals and Treatments

Young (3 months old) and aged (20 months old) wild‐type (WT, C57BL/6) and homozygous global TSPO knock out (TSPO‐KO) mice (Barron et al. 2018) were used for this study comparison. All experiments were carried out in accordance with the National Advisory Committee for Laboratory Animal Research guidelines and approved by the NTU Institutional Animal Care and Use Committee (IACUC# A0384). To induce inflammation, mice were intraperitoneally injected with phosphate‐buffered saline (PBS) or 1 mg/kg lipopolysaccharide (LPS, L2880, Sigma Aldrich) for 4 days. Brains were harvested 24 h post‐injection. Prior to brain harvesting, mice were anesthetized through intraperitoneal injection of 120 mg/kg body weight of sodium pentobarbital and further underwent cardiac perfusion of ice‐cold PBS. Hemibrains were then collected and snap frozen using dry ice cooled 2‐methylbutane and stored at −80°C.

### 2.2 Cell Cultures and Treatments

For primary mouse microglia cultures, postnatal Day 1 WT mice were decapitated, their brains were dissected, and meninges and blood vessels were removed completely. Brains from six pups were harvested and processed for experiments. Tissues from two pups were transferred to 15‐mL tubes and dissociated in 1.5 mL 2.5% trypsin at 37°C for 10 min. Dissociation was terminated by adding an equal volume of complete DMEM (DMEM +10% FBS + 1% Pen‐Strep, DMEM‐COM), followed by 750 μL of 10 mg/mL DNase I. Cells were separated by repeated pipetting and centrifuged at 1200 RPM, 4°C for 10 min. The supernatant was removed, and the pellet was triturated in 1 mL of warm DMEM‐COM (with 0.5 ng/mL GM‐CSF). The cell suspension was added into poly‐L‐lysine (0.1 mg/mL; Sigma Aldrich) coated T75 flask, and cells were left to adhere for 24 h at 37°C, 5% CO_2_. Media was replaced the next day with complete DMEM‐COM (with 0.5 ng/mL GM‐CSF). Cells were grown until a confluent layer was formed without changing the medium to allow microglial proliferation. Microglia were harvested by vigorously tapping the flasks for 5 min. Harvested microglia were plated on a poly‐L‐lysine coated 96‐well glass bottom plate and used in experiments 1 day after plating. For primary bone marrow‐derived macrophage (BMDM) cultures, femur and tibia were collected from aged WT (14 months old) and homozygous TSPO‐KO mice (11 months old) and placed in IMDM + 2% FBS. Epiphyses of femur and tibia were removed, and bone marrow was flushed out onto a 70‐μm nylon cell strainer placed in a 50‐mL tube by slowly injecting 2–3 mL ice‐cold HBSS per bone using a 23G needle and a 10‐mL syringe. The bone marrow dissociated by passing it through the cell strainer with a 5‐mL plunger, followed by washing with 5 mL HBSS. The bone marrow suspension was centrifuged at 200G, 4°C for 5 min, and the supernatant removed. The cell pellet was resuspended with 1 mL of red blood cell lysis buffer (00‐4300‐54, eBioscience) for 5 min at room temperature. The reaction was stopped with 14 mL of IMDM + 2%FBS and centrifuged at 300G, 4°C for 5 min. The cell pellet was resuspended in 12 mL of bone marrow culture media RPMI‐COM (with M‐CSF, 1:50) and seeded into 12‐well plate (1 mL/well). Fresh bone marrow culture media (500 μL) was added on Day 4, and cells were trypsinized and replated onto a 96‐well glass bottom plate for experiment by Day 10.

### 2.3 Senescence‐Associated β‐Galactosidase (SA‐β‐Gal) Assay and NF‐Kβ Immunocytochemistry

Primary microglia or macrophage cultures were pre‐treated with vehicle control, Entinostat 500 nM (MedChemExpress, HY‐12163), or Entinostat with Ro5–4864 (Sigma Aldrich, C5174) for 22 h, followed by co‐treatment with vehicle control or LPS 100 ng/mL for 2 h. To measure SA‐β‐Gal activity, cells were washed with PBS and incubated with senescence dye (Abcam, ab228562) for at 37°C 1 h, followed by two washes with assay buffer. Cells were fixed with 4% PFA for 10 min at room temperature, washed 3× in PBS, then permeabilized with 0.1% Triton X‐100, and blocked in 5% Bovine Serum Albumin (BSA) + 0.2% Triton X‐100 for an hour. Cells were then labeled in primary antibody NF‐kβ (Cell Signaling Technology, 8242) overnight at 4°C, washed 3× in PBS, and then incubated with secondary antibody anti‐rabbit Alexa Fluor 634 (Invitrogen, A31576) for an hour at room temperature. Cells were washed again in PBS for 5 min before adding fluoroshield with DAPI (Abcam, ab104139) diluted with PBS (1:3) in each well.

### 2.4 Imaging and Image Analysis

Images were captured on a Laser Scanning Microscope 800 Zeiss Confocal Setup with a Plan‐Apochromat 63× 1.4NA Oil Objective. Nyquist sampling optimized z‐stack step size (0.34 μm) with twice averaging(mean) option was used. Images were then processed on Imaris 9.9.1 bitplane software (Oxford Instruments). Briefly, images were background subtracted with Gaussian Filter Width based on the smallest sphere object in that channel. Cell Objects were created with each cell consisting of a single nucleus (DAPI channel), and cell demarcation was based on the NF‐Kβ immunoreactivity. Nuclei and cell surfaces were then exported. Heterochromatin foci were identified using baseline subtraction of the DAPI channel. Batch statistics were then calculated from these channels using vantage options. Batch analysis and processing were done across the image analysis, curated with manual inspection to ensure that cells that were either dividing or cut off frame were excluded.

### 2.5 Flow Cytometry Analysis of TSPO in Innate Immune Cells From Mouse Brain

Innate immune cell populations were isolated from mouse brains as previously described (Sheng et al. 2019; Wu et al. 2021). Briefly, hemibrains were first dissociated mechanically and then enzymatically in dissociation buffer (100 mg/mL Collagenase (Roche Applied Science, Basel, Switzerland), 1.2 U/mL Dispase (Roche Applied Science, Basel, Switzerland), and 20 Units/mL DNase I (Life Technologies) in IMDM with 2% FBS) where they were incubated for 30 min at 37°C on a shaker. To achieve a single‐cell suspension, tissue suspensions were passed through a 19‐gauge syringe and a 40‐μm cell strainer. The suspension was then pelleted at 350 g for 5 min at RT and subsequently resuspended in 4 mL 40% Stock Isotonic Percoll (SIP). This Percoll‐cell suspension was then gently overlaid on 3 mL 70% SIP. To isolate the interphase layer, the gradient suspension was then centrifuged for 10 min at 2800 rpm, with deceleration 4 at RT. The interphase layer was then pelleted at 600 g for 5 min and prepared for staining. The staining procedure begins with fixing in 4% PFA, followed by permeabilization with 0.1% Triton X‐100 before staining with antibodies of interest. Blocking of Fc receptor was completed using anti‐CD16/32. The following antibodies were used: CD11c (BD Horizon, clone: N418), CD11b (BioLegend, clone: M1/70), CD45 (BioLegend, clone: 30F11), F4/80 (BioLegend, clone: BM8), MHC II (BioLegend, clone: M5/114.15.2), CD11c (BioLegend, clone: HL3), TSPO (Abcam, 109,497). Flow cytometry data were analyzed with FlowJo software (TreeStar).

### 2.6 RNAseq Data Generation & Analysis

Frozen hippocampi from WT and TSPO‐KO mice (*n* = 4/group, sex‐matched within each group) were used to generate the RNAseq data. A subset of this data, the 3‐month‐old treatment groups, was previously analyzed and published (Fairley et al. 2023). Hippocampi were dissected out of hemibrains at −20°C, homogenized immediately in Trizol, and total RNA extracted. Chloroform (1:5 vol: vol) was added to the Trizol homogenate, then centrifuged at 12,000 rpm for 15 min. The aqueous phase was removed, and total RNA was extracted using the Qiagen RNeasy mini kit column according to the manufacturer's instructions. RNA quality and purity were checked using the Agilent 2100 Bioanalyzer with the Agilent 6000 Nano Kit. Total RNA of the samples had a minimum of RIN 8.3 with an average of RIN 9 and above. Oligo dT mRNAseq stranded library was prepared using the NEBNext Ultra library preparation kit and sequenced using Illumina HiSeqTM.

Quality control of the FASTQ files was performed using FASTQC (http://www.bioinformatics.babraham.ac.uk/projects/fastqc/). Transcripts were quantified and aggregated at the gene level using the Salmon pseudo‐aligner 1.9.0 using the *Mus musculus* genome Ensembl release 99 reference genome. Differential expression analyses were performed using the *DESeq2* (Love et al. 2014) 1.36.0 R package and nominal *p* values were corrected for multiple testing using Benjamini–Hochberg. Log_2_ (fold change) (Log2FC) values were shrunk using the *ashr* 2.2‐54 R package (Stephens 2017) and thresholded at Log2FC ≤ 0.5 or Log2FC ≥ 0.5. Volcano plots of RNAseq were constructed using the *ggplot2* (Wickham 2016) R package 3.5.0 in an R 4.2.3 environment.

For gene set enrichment, the fGSEA R package 1.24.0 (Gennady et al. 2021) was used, ranking genes by the Wald statistic from the differential gene expression analysis. Enrichments were run against the Reactome collection genesets for *Mus musculus* using 10,000 permutations.

### 2.7 RNAseq DEGlist Overlap Analysis

Differentially expressed gene (DEG) lists generated from DESeq2 were compared against each other for significant non‐random associations using the GeneOverlap package (Shen 2023) (version 1.34.0) from Bioconductor. Each DEG list was filtered for significance and either categorized into gene sets as upregulated (FDR < 0.05 and LFC ≥ 0.5) or downregulated (FDR < 0.05 and LFC ≤ −0.5). Fisher's exact test was performed for each gene set where odds ratio represents strength of non‐random association between two gene sets.

### 2.8 Deconvolution Methods for the Estimate of Relative Abundance of Cell Types

Estimation of brain cell type abundance (absolute mode, 100 permutations) was performed using CIBERSORTx (Newman et al. 2019). The reference signature was prepared from single‐cell RNAseq of WT mice hippocampus Zeisel dataset (Zeisel et al. 2015). The count matrix of this dataset was CPM (counts per million) transformed using the Seurat version 4 package (Hao et al. 2024). The bulk mixture of our RNAseq count data was CPM transformed for the deconvolution (same transformation space as the reference signature) using the EdgeR package (Robinson et al. 2010). Permutational univariate ANOVA was used to evaluate the presence of group differences for each cell type (RVAideMemoire version 0.9‐83‐7 package), followed by the Dunn test (rstatix version 0.7.2 package) with FDR correction for post hoc pairwise comparison.

### 2.9 Weighted Gene Co‐Expression Network

For the multiWGCNA (Tommasini and Fogel 2023) analysis, the gene expression count matrix was first normalized with the DESeq2 “variance stabilizing transformation (VST).” WGCNA network was then constructed on the VST transformed data, using soft power threshold of 7, along with the default settings in the other arguments. Eigengenes of each module were extracted for plotting. PERMANOVA (permutational multivariate ANOVA, Euclidean method, 9999 permutations) was performed using the expression data for members of each corresponding module through linear model containing main effects, genotype and age, and interaction term genotype:age as testing covariates. PERMANOVA was performed using vegan package version 2.6‐4. Following PERMANOVA, post hoc pairwise comparisons was performed using estimated marginal means (EMMs) with the same linear model as PERMANOVA. Estimated marginal means was conducted through emmeans package (Searle et al. 1980) version 1.10.0. Module driver genes were identified by high intra‐modular connectivity defined as > 0.9 Pearson's correlation with the module eigengene of that specific module. Using module driver genes as query input, protein–protein interactions within the input was retrieved via Network Analyst platform (Zhou et al. 2019) and STRING database (Szklarczyk et al. 2021) (settings: High confidence PPI > 900/1000 score, zero‐order direct interactions). The final network was pruned using Prize‐collecting Steiner Forest (PCSF) algorithm to isolate high confidence subnetwork consisting of the minimal number of edges.

### 2.10 Transcriptional Motif Analysis

Members of Module 5, the module exclusively upregulated in the TSPOKO aged group, were used as input for findMotifs.pl function in HOMER (Heinz et al. 2010) Motif Analysis. Known enrichment of transcriptional motifs that are either 8 or 10 bp, and −2000 to +500 bp from the transcriptional start site (TSS) of the input genes was identified against all known motifs (29,786 motifs) within the HOMER database. Cognate genes (transcription factors) of significantly enriched (BH < 0.05) motifs were then extracted from the expression matrix. These transcription factors were then integrated as a protein–protein interaction (PPI) network with Module 5 members using Network Analyst software (Zhou et al. 2019) and the STRING database (Szklarczyk et al. 2021).

### 2.11 Connectivity Map Analysis

The Connectivity Map (CMap) database of drug‐induced transcriptional profiles was downloaded from Clue.io (Subramanian et al. 2017) (https://clue.io/data/CMap2020#LINCS2020, updated in 2021). This database is made up of over 720,000 drug perturbation signatures, derived from treating more than 33,000 small molecule compounds on various cell lines, including Neural Progenitor Cell (NPC) and glioblastoma (GI1). The top 100 of the DEGs from the inflammaging comparisons of TSPO‐KO were used as inputs to query the drug CMap database. DEGs were filtered for significance (FDR < 0.05) and are either Log2FC ≥ 0.5 or ≤ −0.5. From within the Library of Integrated Network‐Based Cellular Signatures (LINCS) database, compounded‐treated signatures derived from the NPC (11,195 signatures) and GI1 (2117) cell lines were independently queried with the TSPO‐KO inflammaging signature.

The sscMap algorithm (Zhang and Gant 2008) was utilized to compute the connectivity score, due to its superior sensitivity (Lin et al. 2020; Tham and Langley 2022). The connectivity score can range from −1 to +1, where a positive connectivity score indicates a transcriptional phenocopying effect of a drug with the query signature, while a negative connectivity score indicates a transcriptional reversal of the drug and query signatures. From the individual connectivity scores, signatures treated with the same perturbation ID (pert_id) were summarized by computing their mean connectivity score.

The significance of the score is determined by randomly permuting the gene composition of the query signature 1000 times to generate the null distribution scores. A connectivity score is considered significant if the absolute connectivity score exceeds 99% of the absolute scores from the null distribution.

### 2.12 Nuclear Magnetic Resonance Spectroscopy (NMR) Metabolomics Analysis

Frozen hemibrains from young and aged WT and TSPO‐KO mice (*n* = 4–5/group) were used for NMR analysis. Brain tissues were weighed and subjected to extraction with pre‐cooled methanol:Water (2:1) using a homogenizer (Precellys, France). The supernatant was collected after centrifugation (13,800 g) and the pellet was subjected to a second extraction using the same procedure. The resultant supernatants were pooled, followed by drying using Spin‐Vac (Eppendorf, Germany). Extracted samples were resuspended in 550 μL of D_2_O buffer (pH = 7.4) containing 0.05% sodium 3‐trimethylsilyl (2,2,3,3‐^2^H4) propionate (TSP). Here, TSP is used as a chemical shift reference. NMR spectra were recorded from a Bruker Avance III HD 600 MHz NMR spectrometer (Bruker, Germany) equipped with a 5 mm BBI 600 MHz Z‐Gradient high‐resolution probe. One‐dimensional NMR spectra were recorded using the standard first increment of the NOSEY pulse sequence (recycle delay‐G1‐90°‐t1‐90°‐G2‐tm‐90°‐acquisition) with water suppression both during the recycle delay (4 s) and mixing time (100 ms). A total of 256 scans were collected into 64 k data points with a spectral width of 30 ppm at a temperature of 300 k. A 90° pulse set to ~11 μs and 4 dummy scans were used.

The spectra were manually phased and corrected for baseline distortion and subsequently imported to Amix (Bruker, Germany, version 3.9.15) and the region between 0.5 and 10 ppm was integrated with a 0.003 ppm(1.8 Hz) bucket. The water peak resonance (4.60–4.70 ppm) was removed from each spectrum, and the spectra were normalized to their respective wet weight of brain tissues. The normalized data were then exported to SIMCA (SIMCA‐P15 UMETRICS, Umea, Sweden) for statistical data analysis. Metabolite concentrations were calculated by integration of peak area and further analyzed by univariate analysis.

NMR peak assignment was performed by comparing literature (Abreu et al. 2021; Li et al. 2015; Wang et al. 2005) and some were confirmed with statistical total correlation spectroscopy (STOCSY) (Cloarec et al. 2005). A peak at 4.832 ppm was identified that was only present in the aged TSPO‐KO mice. Since it is a singlet, two‐dimensional NMR is insufficient for the peak assignment. We employed the STOCSY method, which takes advantage of high correlation for peaks in the same molecule or in the same metabolic pathway (Cloarec et al. 2005). It showed high correlation with the peak at 8.462, which is formic acid. The correlation suggests that the peak at 4.832 could be formaldehyde, which is undetectable in normal circumstances. To confirm this assignment, we searched online information. Formaldehyde in water presents the structure of methanediol, and it is a singlet with a chemical shift ranging from 4.4–5.4 ppm (Automated Topology Builder and Repository Version 3.0, Methanediol | CH4O2 | MD Topology | NMR | X‐Ray (uq.edu.au)).

Shifted log (log_10_ *x* + 1) transformation was performed on raw data from targeted NMR due to the right skewness of the data and the presence of multiple zero values. PLSDA (partial least squares discriminant analysis) was conducted using the MixOmics package version 6.22.0 (Rohart et al. 2017). The number of components selected for each corresponding PLSDA was based on the number that gives the least classification error rate for that specified factor (either genotype:age interaction term, main effects genotype or main effects age). The performance of PLSDA was evaluated using 50 repeats and 4‐cross fold validation (folds selection based on 4–5 samples per fold). PLSDA plots were generated using MetaboAnalyst 6.0 (Xia et al. 2009). Permutational univariate ANOVA (999 permutations) with sample groups as covariate, followed by pairwise post hoc Dunn's test, was conducted for statistical comparisons. Statistical analysis of the NMR data was performed in an R environment, R 4.2.3 using the RVAideMemoire package (Hervé et al. 2018) for permutational univariate ANOVA (version 0.9‐83‐7) and rstatix (Kassambara 2023) for Dunn's test (version 0.7.2).

## 3 Results and Discussion

Since TSPO has been implicated in inflammation in brain aging and age‐associated neurodegenerative disease, we investigated the role of TSPO in aging and inflammatory responses in the mouse hippocampus.

We first characterized the effect of aging on hippocampal transcriptional responses, then determined the effect of TSPO deletion under baseline and LPS‐induced inflammatory conditions on hippocampal transcriptional signatures. An interactive shiny app is made to show the overlapping members for each overlapping comparison for the effects of aging, LPS‐induced inflammation, and TSPO deletion (https://keionnbarronlab.shinyapps.io/venn_TSPOaging/).

### 3.1 Aging‐Dependent Transcriptional Signatures Characterized by Inflammation in Mouse Hippocampus

**FIGURE 1:**
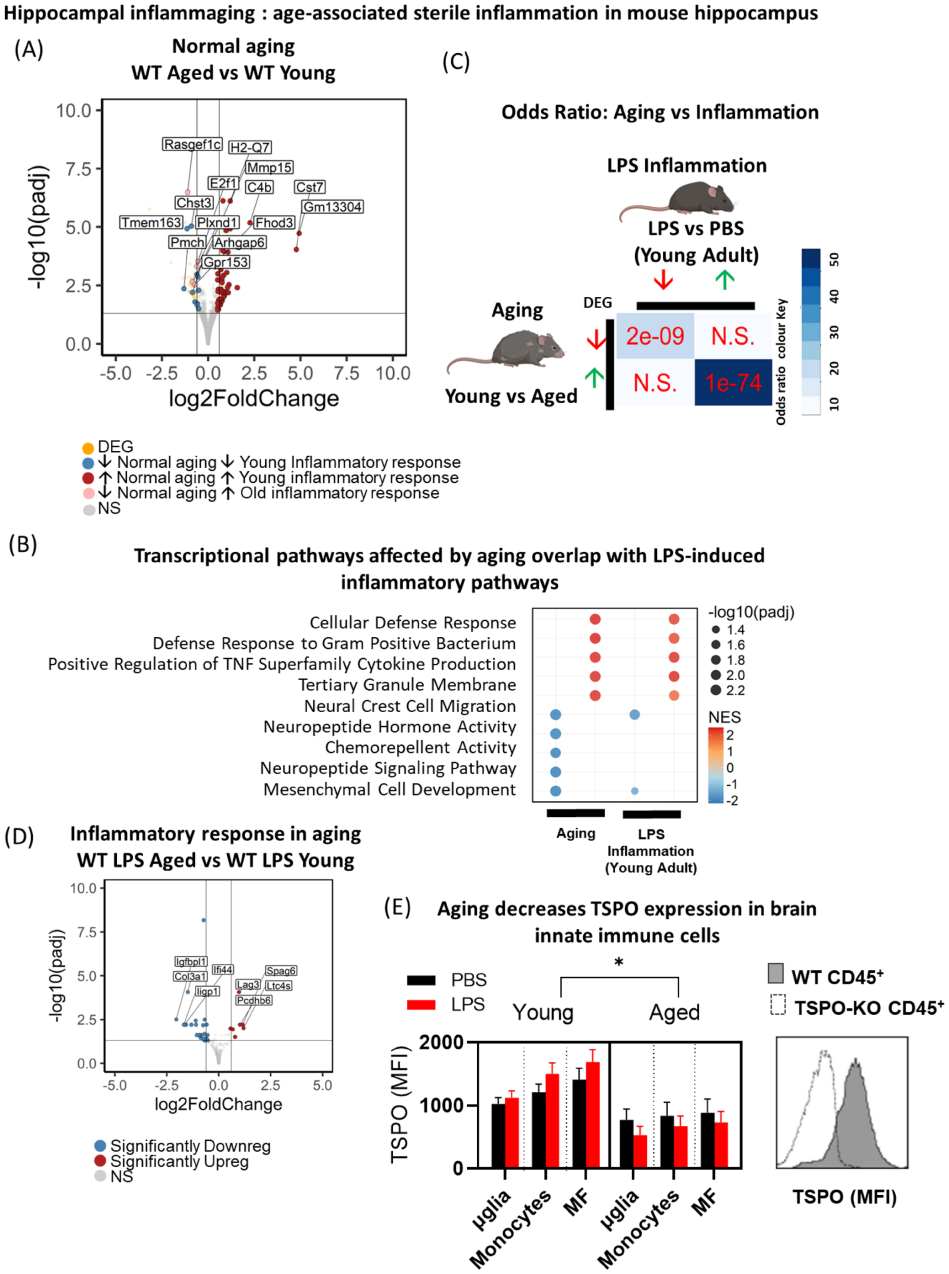
Hippocampal inflammaging: Age‐associated sterile inflammation in mouse hippocampus. (A) Volcano plot showing differentially expressed genes in normal aging hippocampus (aged WT PBS versus young WT PBS). Overlapping differentially expressed genes (DEGs) between this comparison and inflammatory response in the young comparison (young WT LPS vs. young WT PBS) are identified. Specifically, overlapping DEGs that are downregulated in both comparisons are blue, while upregulated DEGs are in red. (B) Comparison of overlapping gene set enrichment pathways between normal aging and inflammatory response in the young. (C) Hypergeometric testing of DEG overlap between normal aging and inflammatory response in the young. Odds ratio represents strengths of positive association between the two DEG sets. Magnitude of odds ratio is represented by color key (blue palette) while *p* value scoring is labeled (red) (D) Volcano plot showing differentially expressed genes in inflammatory aging hippocampus (aged WT LPS versus young WT LPS). (E) *Left:* Flow cytometry quantification of TSPO protein levels in innate immune cells isolated from brain (MFI, median fluorescence intensity). Microglia (μglia): CD45^hi^F4/80^hi^cd11B^+^; monocytes: CD45^int^F4/80^hi^cd11B^+^Ly6c^+^MHCII^−^; monocyte‐derived macrophages (MF): CD45^int^F4/80^hi^cd11B^+^Ly6c^+^MHCII^+^. *Right*: Representative histogram of TSPO fluorescence in CD45^+^ cells isolated from WT and TSPO‐KO mouse brain. Statistical tests: 2E: Three‐way ANOVA with FDR post hoc. *p* < 0.05.

We determined normal age‐related transcriptional signatures in the hippocampus by comparing the hippocampal transcriptome between young adult (3 months) versus aged (18 months old) WT mice (WT: 167 DEGs, 96 upregulated, 71 downregulated; FDR < 0.05, shrunken LFC > shrunken LFC ≥ 0.5 or ≤ −0.5; Figure 1A; Table S1). Functional enrichment analysis indicated aging was associated with increased inflammatory transcriptional signatures, where top pathways with upregulated genes included cellular defense response, defense response to gram‐positive bacterium, positive regulation of TNF superfamily cytokine production (Figure 1B, Tables S2 and S3). Top pathways with genes downregulated in aging included mesenchymal cell development and chemorepellent activity, which predominantly reflected an abundance of genes encoding semaphorins (*Sema3a,c,d,f,g,4a,c,g,5b,6a,b,7a*). Semaphorins play critical roles in adult hippocampal connectivity, plasticity, myelination, and neurogenesis (Carulli et al. 2021; Sebastian et al. 2018). Neuropeptide signaling pathway genes were also downregulated in aging, including neuropeptides involved in hippocampal learning and memory (*Ntsr1/2*) (Li et al. 2013; Yamauchi et al. 2007), synaptic plasticity and neurotransmission (*Gal*, *Adcyap*, *Nrg1*, *Sstr1/2/5*, *Tenm1*) (Cabezas‐Llobet et al. 2018; Coumis and Davies 2002; Honoré et al. 2021; Johnson et al. 2020; Kondo et al. 1997; Shamir et al. 2012), and stress responses (*Cartpt*, *Crh*, *Avp*, *Pnoc*, *Oprm1*, *Oprk1*, *Pmch*) (Hunter et al. 2007; Maras and Baram 2012; Shi et al. 2020; Zagrean et al. 2022).

The hippocampal age‐associated inflammatory response (inflammaging) partially overlapped with the inflammatory transcriptional response induced by LPS in the young adult hippocampus (65/125 aging‐associated upregulated DEGs overlapped with upregulated DEGs following LPS treatment, *p* < 0.0001, odds ratio = 56.5; Figure 1C, Table S4). These included genes involved in innate immune responses (*Nlrp3*, *Ifi204*, *Ifit3*, *Tlr2*), complement cascade (*C4b*, *C1qa/b/c*, *C1ra*, *C3*, *C3ar1*), and antigen presentation (*H2‐D1/K1/Q6/7*, *Ncf1/2*). Reflecting this, top enriched age‐associated functional pathways were also pathways associated with LPS‐induced inflammation (Figure 1B, Table S5). This is consistent with previous studies that have described increases in antigen presentation and complement pathways in the aging brain (Cribbs et al. 2012; VanGuilder et al. 2011), hypothesized to be associated with increased priming of innate immune responses and microglial tolerance and senescence in the aged brain (Costa et al. 2021).

LPS is widely used as a model for age‐ and disease‐associated inflammation, since it induces cognitive impairment and mimics both neuropathological and cognitive changes observed in dementia and delirium (Ang et al. 2023; Bahaidrah et al. 2022; Brown and Heneka 2024; Kealy et al. 2020; Nishiguchi et al. 2024; Sultan et al. 2021; Zhang, Ma, et al. 2021). This may reflect common inflammatory transcriptional pathways upregulated in aging and by LPS, as found in the current study. Inflammation can impair critical processes such as neurogenesis, plasticity, and myelination, which are vital for hippocampal function, contributing to age‐and LPS‐induced cognitive impairment (Cammarota and Boscia 2023; Perez‐Dominguez et al. 2019; Valero et al. 2014).

Interestingly, in the aged brain, we observed a minimal effect of LPS on transcription signatures measured by differential expression analysis (aged LPS vs. aged vehicle‐treated mice: 9 DEGs; 7 upregulated, 2 downregulated; FDR < 0.05, shrunken LFC ≥ 0.5 or ≤ −0.5; Table S6). Likewise, we observed a low number of differentially expressed genes between LPS treated young and aged mice (young LPS vs. aged LPS: 34 DEGs, 8 upregulated, 26 downregulated; Figure 1D, Table S7). This suggested that the heightened inflammatory state in the aged hippocampus may limit further transcriptional responsiveness to LPS, as compared to the young adult, where a robust immune transcriptional response was detected relative to the low baseline levels of inflammation.

### 3.2 TSPO Levels Decline in Innate Immune Populations in Aging Mouse Brain

To determine the effect of aging on TSPO expression in innate immune populations in the brain, we measured TSPO levels by flow cytometry in microglia, monocytes, and macrophages isolated from young and aged mouse brains. Intriguingly, despite heightened inflammatory signatures in the aged hippocampus, TSPO expression decreased in all three innate immune cell populations with aging (*n* = 6–10 mice/group, three‐way ANOVA, age main effect: *F* = 26.84, *p <* 0.0001; Figure 1E). Additionally, the effect of LPS treatment on TSPO expression was age‐dependent, as indicated by a significant age × LPS treatment interaction (*F* = 3.62, *p* = 0.04). While TSPO is widely regarded to increase in the aging brain due to inflammaging (e.g., Repalli 2014), contrary to expectations, our findings show that TSPO protein expression declines in the innate immune cells of the aging mouse brain. This discrepancy may arise because most studies reporting an age‐associated increase in TSPO have relied on in vivo PET imaging (Gulyás et al. 2011; Paul et al. 2019; Schuitemaker et al. 2012), which cannot differentiate between cell‐type–specific contributions to TSPO signals. Notably, endothelial cells are a strong contributor to TSPO‐PET signals (Wimberley et al. 2021). Meanwhile, other studies in human cohorts report region‐specific elevations in TSPO signals, restricted to hypothalamic and thalamic regions (Butler et al. 2022; Cagnin et al. 2001; Kumar et al. 2012), while yet others have found no association between brain TSPO‐PET signals and aging (Suridjan et al. 2014). In mouse models of AD, TSPO‐PET signals in various brain regions, including the hippocampus, exhibit a U‐shaped trajectory, peaking midlife before gradually declining with further aging (Brendel et al. 2017; Focke et al. 2019). LPS has been widely shown to induce TSPO expression in mouse microglia, monocytes, and macrophages (Blevins et al. 2021; Nutma et al. 2023; Shimoyama et al. 2019) and in humans, increased TSPO‐PET signals are detected following LPS administration (Sandiego et al. 2015). However, our findings indicate that the effect of LPS on TSPO expression in innate immune cells may also be age dependent. Together, our findings challenge the prevailing notion that TSPO expression increases in brain innate immune populations with aging and highlight the importance of cell‐type–specific contributions, regional variability, and methodological consideration when interpreting TSPO as a marker of inflammation in the context of aging.

### 3.3 Hippocampal Inflammaging Is TSPO Independent

**FIGURE 2:**
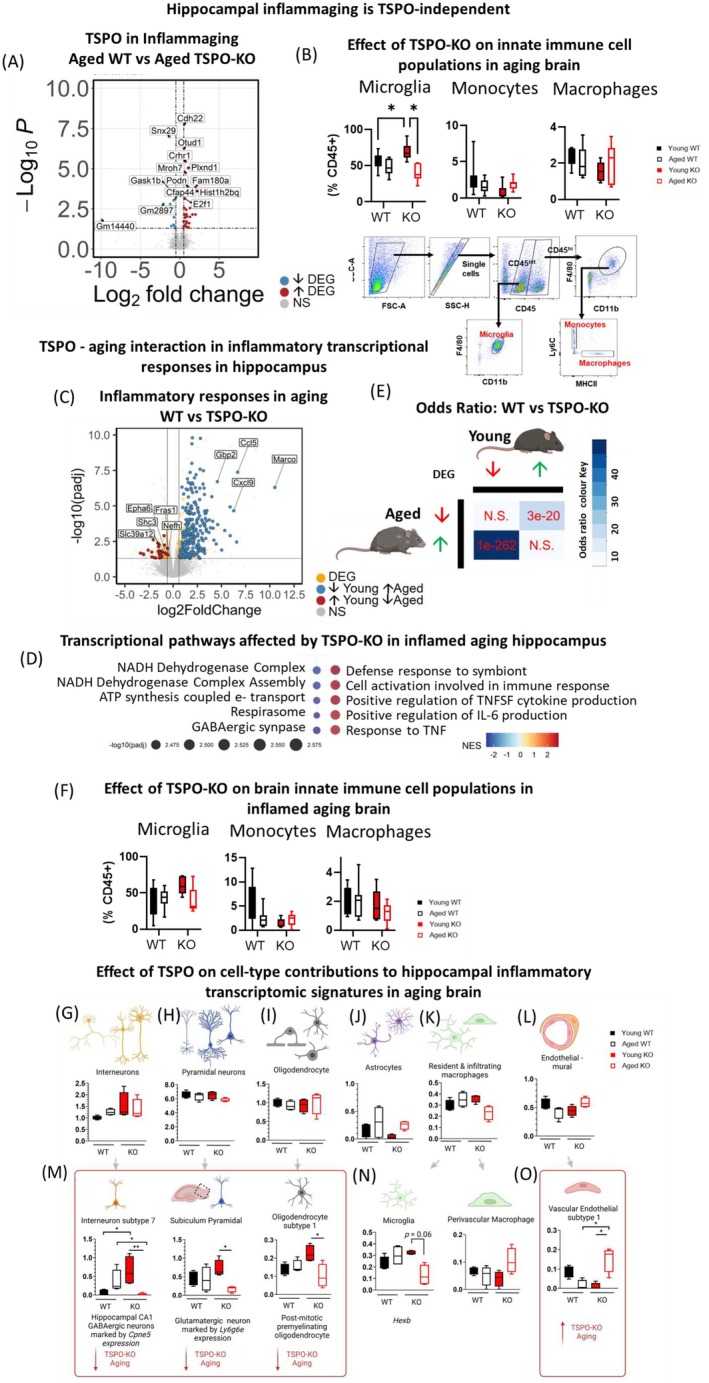
TSPO–aging interaction in inflammatory transcriptional responses in mouse hippocampus. (A) Volcano plot showing differentially expressed genes in aged WT versus aged TSPO‐KO hippocampus at baseline. Differentially expressed genes (DEGs) were either coded in red (significantly upregulated) or blue (significantly downregulated). Significant DEGs are thresholded at FDR < 0.05, with upregulation thresholded at Log2FC ≥ 0.5 and downregulated at Log2FC ≤ −0.5 respectively. (B) *Top:* Effect of TSPO deletion on proportions of brain innate immune cell populations across normal aging. Bottom: Representative gating strategy. Microglia (μglia): CD45^hi^F4/80^hi^cd11B^+^; monocytes: CD45^int^F4/80^hi^cd11B^+^Ly6c^+^MHCII^−^; monocyte‐derived macrophages: CD45^int^F4/80^hi^cd11B^+^Ly6c^+^MHCII^+^. (C) Volcano plot showing differentially expressed genes in aged WT versus aged TSPO‐KO hippocampus following LPS injections. Significant DEGs were thresholded at FDR < 0.05 an indicated as orange. DEGs that were reversed in aged compared to young WT versus TSPO‐KO hippocampus indicated in blue (significantly downregulated in young but upregulated in aged) and red (significantly upregulated in young, but downregulated in aged) respectively. (D) Fast gene set enrichment analysis (fGSEA) of DEGs in aged WT versus aged TSPO‐KO hippocampus following LPS treatment using Gene Ontology Biological Process pathways. Top 5 up‐ and down‐regulated enriched pathways ranked by normalized enrichment score (NES). (E) Comparison of DEG overlap between aged and young WT versus TSPO‐KO comparisons using hypergeometric testing. Odds ratio represents strengths of positive association between two DEG sets. Magnitude of odds ratio is represented by color key (blue palette) while *p* value scoring is labeled (red). (F) Effect of TSPO deletion on proportions of brain innate immune cell populations isolated from inflamed aging brain measured by flow cytometry. Representative gating strategy shown in (B). (G–O) Estimation of hippocampal cell‐specific contributions to transcriptional signatures detected in LPS treated WT and TSPO‐KO mice using CIBERSORTx. Units of the graphs are arbitrary. Data shown as median, interquartile range with error bars indicating minimum and maximum. Statistical tests: A, C: Benjamini–Hochberg (BH) corrected FDR < 0.05; NS, non‐significant. B: Two‐way ANOVA with FDR post hoc *p < 0.05. E: Fisher's Exact test to test. F: Two‐way ANOVA. G–O: Permutational univariate ANOVA, Dunn's multiple comparison post hoc test. **p < 0.01. *p < 0.05. Created in BioRender. https://BioRender.com/v05x181.

To determine if age‐dependent loss of TSPO could play a role in brain inflammaging, we investigated if genetic TSPO deletion alters age‐associated inflammatory signatures. Our comparison of transcriptional signatures in the hippocampus of aged WT and TSPO‐KO mice revealed surprisingly little effect of TSPO‐KO on transcription, with only 34 DEGs (24 upregulated, 10 downregulated; FDR < 0.05, shrunken LFC >shrunken LFC ≥ 0.5 or ≤ −0.5 Figure 2A; Table S8). This suggested age‐associated hippocampal inflammation is largely TSPO independent.

Measurement of innate immune cell populations isolated from the brain and quantified by flow cytometry revealed increased total brain microglia in young adult TSPO‐KO mice, which were depleted age dependently (*n* = 6–10 mice/group Two‐way ANOVA, age × genotype interaction: *F* = 12.20, *p* = 0.026; FDR post hoc: WT young vs. KO young *p* = 0.04, KO young vs. aged *p* = 0.0006; Figure 2B). In WT mice, aging had no significant effect on microglial numbers (WT young vs. aged *p* = 0.63). In contrast, no effect of aging or TSPO deletion was observed on brain populations of infiltrating monocytes or monocyte‐derived macrophages (monocytes two‐way ANOVA, age main effects: *F* = 0.21, *p* = 0.81, genotype main effects: *F* = 6.11, *p =* 0.19; macrophage two‐way ANOVA age main effects: *F* = 0.49, *p* = 0.71, genotype main effects: *F* = 5.88, *p* = 0.21; Figure 2B). These findings suggest TSPO deletion may result in an age‐dependent impairment of microglial proliferative capacity, which could reflect increased cellular senescence (Hayakawa et al. 2007; Miller and Streit 2007; Sharaf et al. 2013; Streit et al. 2008; Tremblay et al. 2012).

Given that TSPO‐ and age‐dependent effects were observed on microglial numbers, we next applied statistical deconvolution methods to estimate the cell‐type–specific contributions to age‐associated transcriptional signatures from the hippocampal bulk transcriptomic data. These methods leverage reference gene expression profiles for various cell types in the mouse brain to estimate the relative proportion of the transcriptional signatures attributable to specific cell types. No changes in estimates of contributions of major hippocampal cell types was detected between WT and TSPO‐KO conditions (interneurons, pyramidal neurons, oligodendrocytes, macrophages or endothelial‐mural cells; Figure S1). However, aging was associated with a significant decline in the estimated contribution of astrocytes, a glutamatergic pyramidal neuron subtype found in the subiculum (subiculum subtype), and post‐mitotic pre‐myelinating oligodendrocyte subtype (subtype 1) (astrocytes, main effect: age, *F* = 5.08, *p* = 0.04; interneuron subtype 15, main effect: age, *F* = 10.81, *p =* 0.006; oligodendrocyte subtype 1, main effect: age, *F* = 15.10, *p* = 0.005; subiculum pyramidal neurons, main effect: age, *F* = 5.45, *p =* 0.04; Figure S1). These findings indicate that although TSPO deletion leads to age‐dependent alterations in microglial numbers, it does not appear to drive broader microglia‐mediated changes in hippocampal transcriptional signatures.

### 3.4 TSPO–Aging Interaction in Inflammatory Transcriptional Responses in the Hippocampus

Next, we examined the effect of TSPO deletion on LPS‐induced transcriptional responses in the context of aging. TSPO deletion was associated with robust transcriptional differences in the hippocampal inflammatory response in aged mice, with 1184 significant DEGs identified in aged TSPO‐KO mice treated with LPS (FDR < 0.05; Figure 2C, Table S9). Of these DEGs, 593 were upregulated and 182 were downregulated genes with a shrunken Log2FC of either ≥ 0.5 or ≤ −0.5, Figure 2C, Table S9). Gene set enrichment indicated a downregulation of genes belonging to pathways related to the GABAergic synapse, respirasome, ATP synthesis coupled electron transport, and NADH dehydrogenase complex assembly, and an upregulation of genes in pathways involved in immune responses, including defense response to symbiont, cell activation involved in immune response, positive regulation of tumor necrosis factor (TNF) superfamily and interleukin (IL) IL‐1β production, and response to IL‐6 production (Figure 2D, Table S10). This was surprising since, in the young adult mouse hippocampus, we found the opposite effect of TSPO deletion on inflammatory responses, including a dampening of responses to TNF signaling (Fairley et al. 2023).

We directly tested the overlap between TSPO‐KO–specific transcriptional signatures in the young (Fairley et al. 2023) versus aged inflammatory hippocampus. Aging resulted in a reversal of TSPO‐KO transcriptional signatures following inflammatory insult. A significant overlap between young *upregulated* and aged *downregulated* DEGs (*p* < 0.0001, odds ratio = 11.39; Figure 2E, Table S11), and young *downregulated* and aged *upregulated* DEGs was observed in the inflammatory TSPO‐KO hippocampus (*p* < 0.0001, odds ratio = 46.10; Figure 2E, Table S11). Functional annotation indicated genes that were upregulated in the young but downregulated in the aged inflammatory TSPO‐KO hippocampus related to transmembrane and calcium ion transport and myelination. Meanwhile, genes that were downregulated in the young but upregulated in the aged inflammatory TSPO‐KO hippocampus related to immune system processes, including phagocytosis, respiratory burst, and superoxide production, complement pathway, cellular response to interferons (β, γ), regulation of TNF production, and NF‐kβ signaling. This indicated an interaction between TSPO and aging in inflammatory responses.

### 3.5 TSPO–Aging Interaction Changes Heterogeneity of Hippocampal Cell‐Type Contributions to Inflammatory Transcriptomic Signatures

To determine whether changes in innate immune cell composition, such as increased immune cell infiltration, contributed to the TSPO–aging interaction in inflammatory transcriptional responses, we quantified microglial, monocyte, and monocyte‐derived macrophage populations in the brains of young and aged LPS‐stimulated WT and TSPO‐KO mice. No significant effect of TSPO deletion or aging was observed on brain innate immune cell populations (*n* = 5–12 mice/group; Figure 2F). Of note, in contrast to baseline conditions, TSPO‐KO did not affect microglial numbers under inflammatory conditions in either the young or aged mice. These findings suggest that the TSPO–aging interaction in inflammatory transcriptional signatures may not be driven by differences in peripheral immune cell infiltration or microglial proliferation. However, our analysis of innate immune cell populations does not account for region‐specific changes or the heterogeneity of microglial and macrophage subtypes, which may contribute to the observed transcriptional differences in aged‐TSPO‐KO mice.

To better understand this in the hippocampus, we further investigated cell‐type specific contributions to the TSPO–aging interaction in inflammatory transcriptional signatures of the hippocampus using computational deconvolution methods. No changes in the estimated contribution of the major hippocampal cell populations were detected (interneurons, pyramidal neurons, oligodendrocytes, astrocytes, macrophages or endothelial‐mural cells; Figure 2G–L). However, an age‐related decrease in the estimated contribution of a subtype of GABAergic interneurons (subtype 7) found in the hippocampal CA1 region, a glutamatergic pyramidal neuron subtype found in the subiculum (subiculum subtype), and a post‐mitotic pre‐myelinating oligodendrocyte subtype (subtype 1) was detected in LPS‐treated TSPO‐KO but not WT hippocampus (Figure 2M; interneuron subtype 7: main effect: age × genotype, *F* = 28.44, *p* = 0.004; Dunn's multiple comparison *p* = 0.009; pyramidal neuron subiculum subtype: age × genotype, *F* = 4.50, *p* = 0.05, Dunn's post hoc corrected for multiple comparisons young vs. old TSPO‐KO *p* = 0.02; oligodendrocyte subtype 1: age × genotype *F* = 8.53, *p =* 0.008, Dunn's post hoc corrected for multiple comparisons young vs. aged TSPO‐KO *p* = 0.045). These findings suggest that TSPO deletion may specifically impact hippocampal neuronal and oligodendrocyte subtypes, potentially contributing to the downregulation of GABAergic synapse‐related genes and the age‐related decrease in myelination‐related genes identified in aged TSPO‐KO mice under inflammation (Figure 2C).

With respect to the immune cells, estimated microglial contributions revealed a decrease in a microglia subtype characterized by expression of *Hexb* in LPS‐treated, aged TSPO‐KO mice (age × genotype *F* = 12.85, *p* = 0.002, Dunn's post hoc corrected for multiple comparisons *p* = 0.06; Figure 2N). While it did not reach significance after correction, this was surprising given that microglia are a key inflammatory mediator in the brain, and TSPO‐KO was associated with severely exacerbated transcriptional inflammatory signals in aging. The gene signature of this microglial subtype was enriched for pathways involved in cytokine–cytokine receptor interaction, osteoclast differentiation, and phagosome (Table S12). Likewise, estimates of perivascular macrophage contributions did not significantly change across treatments (age × genotype *F* = 16.15, *p* = 0.059). However, interestingly, genes identifying a proangiogenic subtype of perivascular macrophages (subtype 2) were detected exclusively in aged TSPO‐KO mice under inflammation. This was coupled with an increase in contributions of vascular endothelial cells subtype 1 (age × genotype *F* = 28.99, *p* = 0.002, Dunn's posthoc multiple comparisons TSPO‐KO aged vs. young *p* = 0.02, WT aged vs. TSPO‐KO aged *p* = 0.03), which was enriched with genes involved in cytokine–cytokine receptor interaction pathways (Figure 2O). Both the perivascular macrophage and the vascular endothelial subtype that were increased in the TSPO‐KO in inflammaging were enriched in cytokine–cytokine receptor interaction pathways. Analysis of the Zeisel dataset (Zeisel et al. 2015) indicated that these perivascular and endothelial populations express high levels of TSPO in adult wild‐type hippocampus, even compared to microglia, although their relative estimated abundance was far lower than microglia. This is in concordance with our analysis of TSPO protein expression, where macrophages were enriched with TSPO levels compared to microglia, although they represent < 5% of all brain CD45+ cells. Upregulated TSPO expression in perivascular macrophages has been shown in a number of neuroinflammatory conditions, including acute hemorrhagic leukoencephalopathy, viral infection, and cerebrovascular disease (Victorio et al. 2024). In the context of cerebrovascular disease, TSPO upregulation in perivascular macrophages occurred independently of microglia (Nutma et al. 2023; Wright et al. 2020), suggesting that TSPO expression and function may potentially be regulated in an immune cell type and disease‐specific way. Future studies could address the cell‐specific immune functions of TSPO in these cell populations using conditional knockout mouse models.

### 3.6 TSPO–Aging Interaction Linked to Transcriptional Control of NF‐kβ and Interferon Regulatory Factors (IRFs)

**FIGURE 3:**
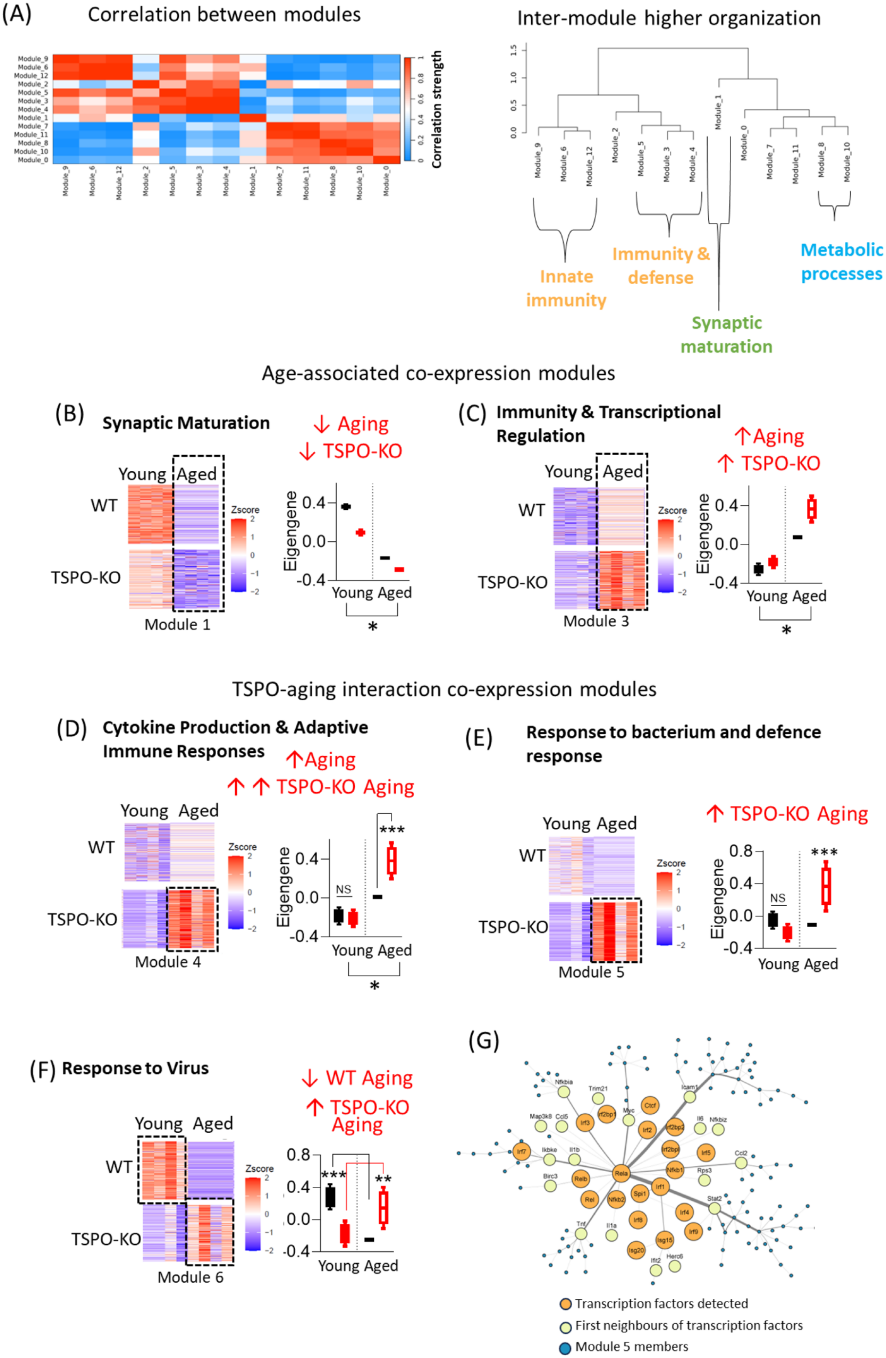
Multi WGCNA reveals co‐expression network associated with TSPO in aging and inflammaging. (A) Heatmap (*left*) and cluster dendrogram (*right*) showing inter‐module relationship among the modules detected. Representation of individual module is defined by the module eigengenes (first principal component, PC1, of each module). Heatmap is calculated from the pairwise correlation between every module eigengene, with intensity as strength of correlation. Hierarchical clustered dendrogram shows eigengene networks, which comprises of one/multiple module eigengenes. (B–E) Differential expression of modules functionally characterized by gene set enrichment analysis as (B) synaptic maturation module, (C) transcriptional and translational regulation module, (D) cytokine production & adaptive immune responses module, (E) response to bacterium and defense response module, and (F) response to virus module. For each module heatmap of expression of module members for young and aged WT and TSPO‐KO samples shown (*left*) and graph of module expression summarized by the module eigengene for each group shown (*right*). Module expression, summarized by the module eigengene, or the PC1, is shown for each group. Eigengene data boxplots are inclusive of its median and interquartile range with error bars indicating minimum and maximum. (G) Integrated transcription factor–module members network of Module 5 (module exclusively upregulated by TSPO‐KO Aged group). Edges refer to protein–protein interactions retrieved via STRING database. Orange nodes are the transcription factors whose motifs are significantly enriched (*BH p‐adjusted value* < 0.05) using Module 5 members as the input list to HOMER software. Yellow nodes refer to first neighbors of the transcription factors within Module 5. Transcription factors which have the highest degree connectivity (protein–protein interactions with the rest of Module 5) *NF‐Kβ* (e.g., *Rela*) and interferon transcription factors families (e.g., *Irf1*, *Isg15*). Statistical tests: A–F: Multivariate PERMANOVA, EMM post hoc test. *p < 0.05. **p = 0.02. ***p < 0.002.

To describe systems‐level transcriptional variation and identify discrete gene co‐expression patterns associated with aging and TSPO deletion, we used a multi weighted correlation network analysis (multiWGCNA). This approach allows us to identify genes which cluster by their expression patterns across datasets with a temporal aspect. These clusters are termed gene co‐expression modules. Gene co‐expression provides insight into relationships between genes that can be used to identify regulatory networks, biological pathways, and processes. WGCNA has proven to be a powerful method for identifying regulatory networks and functional pathways linked to biological processes such as aging (Holtman et al. 2015; Langfelder and Horvath 2008). In our dataset, thirteen gene co‐expression modules were identified (Figure 3A, Table S13). The relationship between these co‐expression modules was evaluated by examining the inter‐module higher organization, with strongly correlated modules organized into a network based on connectivity (Figure 3A). Module interconnectivity suggests coordinated coregulation of modules. Of these, Modules 6, 9 and 12 exhibited high connectivity within a network, as did Modules 3–5 and 0, 7, 8, 10 and 11; suggesting coordinated co‐regulation of these three subnetworks of modules. Gene Ontology overrepresentation analysis identified enriched functional terms associated with 9 of the modules within the network (Modules 1, 3–6, 8–10, 12; Figure 3A; Table S14). Module 1, which had low connectivity in the network suggesting independent regulation and function of this module, was functionally associated with synaptic maturation. Modules 6, 9 and 12, which exhibited a high degree of interconnectivity in the network, were enriched for terms related to immune function and responses, particularly innate immune response. Modules 3–5, which formed a separate subnetwork with a high degree of interconnectivity, were also enriched for immune‐related functions. Modules 8 and 10, which were in the third interconnected subnetwork, were both functionally related to metabolic function. No significantly enriched functional terms were identified associated with Modules 0, 2, 7, or 11.

To identify which of these modules within the network were associated with aging and/or TSPO deletion, we then used factorial PERMANOVA analysis of multivariate gene expression for each module (Tommasini and Fogel 2023). This approach enables module–multi‐trait association for complex experimental design (main effects: age, genotype, age × genotype interaction). Six modules significantly differed across age and/or genotype (Modules 1–6; Table S13). This was reflected in the module‐trait associations described below.

Modules 1 and 3 were age‐associated co‐expression modules. Module 1 was functionally associated with synaptic maturation and contained 7724 downregulated genes in both aging and TSPO‐KO conditions (PERMANOVA: main effect age: *p =* 0.0001, genotype *p* = 0.0004, genotype × age *p* = 0.0032; Tables S13 and S14, Figure 3B). This was consistent with previous aging studies showing age‐associated downregulation of pathways related to synaptic maturation, plasticity, and function in aging, which is thought to be a key factor in age‐related cognitive decline (Smith et al. 2020). Interestingly, TSPO deletion exacerbated this age‐associated depletion of synaptic transcriptomic signature in both young and aged mouse hippocampi.

Conversely, Module 3, containing 1783 genes, was functionally associated with immunity and transcriptional and translational regulation, and was upregulated in both aging and TSPO‐KO conditions (PERMANOVA: main effect age: *p* = 0.004, main effect genotype: *p =* 0.0002; Figure 3C). Immune‐related functional terms associated with Module 3 included innate immunity and myeloid differentiation. These immune functions likely involved integrin–GTPase associate cell adhesion signaling processes, since *Itgb1* and *Cdc42* were identified as hub genes with the most protein–protein interactions (PPI) within a network constructed from the genes with high intra‐module connectivity (module driver genes) (Figure S2). Previous aging studies have also identified increased immune activation as a key signature in multiple regions of the aging brain (Ham and Lee 2020). Meanwhile, transcriptional and translational regulatory functional terms in Module 3 highlighted transcriptional elongation, regulation of RNA polymerase II promoter, and ribosome and ribonucleoprotein complex biogenesis (Table S14). Previous studies have shown age‐related changes in transcriptional elongation and RNA splicing are associated with longevity across multiple organisms across different tissues, including brain, and that mutations that alter RNA polymerase II activity increase longevity (Debès et al. 2023). Within the PPI network, ribosomal genes involved in cell stress, cell cycle arrest, and DNA damage were identified as hub genes, such as *Rps27a*, which has been identified as an early indicator of cell stress (Figure S2). Since these immune and transcriptional genes were identified as part of the same coregulated network, one possible explanation is that these age‐related changes in immunity and transcription/translation are causally linked. Supporting this notion, several potential module driver genes were found to act as dual members of immune and transcriptional regulatory functional terms. These genes included *Lpxn*, *Zfp36*, *Lgals3*, *Elf1*, and *Cd33*. Similar to observations in the synaptic maturation module, TSPO deletion exacerbated these age‐related transcriptomic signatures of Module 3, increasing expression in both young and aged mouse hippocampi. These findings indicate TSPO deletion exacerbates some synaptic and immune age‐related transcriptional changes in the hippocampus.

Modules 4–6 were associated with the TSPO–aging interaction. Module 4, containing 443 genes, was identified as an immune module, associated with cytokine production and adaptive immune responses, and like Module 3, the module genes were also upregulated in aging (PERMANOVA: main effect age: *p =* 0.0004; Table S14). Supporting the functional enrichment results, a PPI subnetwork of immune‐related transcription factors and helicases including *Jak1/2, Stat3*, and *Rela* was identified using the STRING database (Figure S3). *JAK/Stat* is involved in cytokine signal transduction and response (e.g., interferons), while *Rela* is a transcription factor critical in *NF‐kβ* activation (Yu et al. 2004). Interestingly, this age‐related increase in expression in Module 4 was markedly exacerbated in the TSPO‐KO condition (age × genotype: *p* = 0.0045, EMM post hoc aged WT s aged TSPO‐KO *p* = 0.00013; Figure 3D, Table S13). Meanwhile, another immune module (Module 6, 223 genes), involved in viral responses and innate immunity, was downregulated in aging in WT but upregulated in aging in the TSPO‐KO hippocampus (PERMANOVA: age × genotype *p* = 0.0001; EMM post hoc: WT young vs. old *p =* 0.001, TSPO‐KO young vs. old *p =* 0.018; Figure 3F, Tables S13 and S14). Immune pathways associated with Module 6 included antigen processing and presentation, phagocytosis, necroptosis, regulation of NF‐kβ signaling, and response to interferons. Previous studies have demonstrated TSPO‐PET can be used to visualize immune responses to viral infections (Shah et al. 2022). Our data suggest TSPO may play a role in dampening viral immune responses in the aging hippocampus, and that this TSPO sensitivity to viral infections may be regulated by receptors involved in antigen presentation, given that the top five members with the highest intramodulatory connectivity for Module 6 were *Klhl21*, *Clec4a3*, *Gbp2*, *Trim30a*, and *Iigp1* (Table S15).

An immune module that was functionally associated with response to bacterium and regulation of defense response (Module 5, 371 genes) was of the highest interest among the coregulatory network because its genes were exclusively upregulated within the aged TSPO‐KO group (PERMANOVA: age × genotype *p* = 0.0001; TSPO‐Figure 3E, EMM post hoc aged TSPO‐KO vs. all other groups *p* < 0.002; Tables S13 and S14). This module, like Module 6, was enriched in genes functionally involved in pathways that were downregulated in young but upregulated in aged TSPO‐KO hippocampus. This included phagocytosis, respiratory burst and superoxide generation, TNF production, and NF‐kβ signaling. To predict potential transcription factors involved in regulating this TSPO–aging interaction, we leveraged transcription factor binding motifs in promoters (−500 to +2000 bp from TSS) based on the genes in this module (Module 5; Figure 3G). Nine transcription factor motifs were significantly enriched (FDR < 0.05; Table S16). A total of 20 cognate genes (transcription factors) of these motifs were found within the expression matrix. These were integrated as a protein–protein interaction network with Module 5 members (Figure 3G). The *NF‐kβ* transcription factor, *Rela*, was identified as the hub node based on the highest degree of connectivity (21 protein–protein interactions) and shortest average path (3.027) within the network (Table S16). *Rela* was also identified as a hub in the PPI network of Module 4, suggesting some overlapping regulatory pathways between these two modules. Additionally, most of the mapped transcription factors (13/20) in module 5 belonged to the interferon transcription factors family (e.g., *Irf1*, *Isg15*). Interestingly, *Spi1*, a macrophage–microglia transcription factor (Zhang et al. 2024), is predicted to interact with enriched interferon transcription factors (*Irf1*, *Irf4*, *Irf8*), suggesting that macrophage and/or microglia may be involved in mediating the interferon signaling network seen in Module 5 (Figure 3G). Together, the integrated network of transcription factor–Module 5 indicates that the immune pathways enriched within Module 5 may be largely mediated by *NF‐kβ* and interferon signaling. NF‐kβ signaling was commonly identified between immune Modules 4, 5, and 6. Our results indicate a link between TSPO and *NF‐kβ* is not an isolated finding. A previous study also identified NF‐κB as a key signaling pathway mediating transcriptional changes following TSPO deletion in Leydig cells (Fan and Papadopoulos 2021). Similarly, Desai et al. (2020) demonstrated that TSPO promotes NF‐kβ activity by facilitating cholesterol redistribution to the nucleus through retrograde mitochondrial‐nuclear signaling in breast cancer cells. Conversely, Da Pozzo et al. (2019) found that NF‐kβ itself regulates TSPO expression, with a binding site identified in the *Tspo* promoter, suggesting a regulatory feedback loop, where elevated NF‐kβ activity upregulates TSPO expression, which in turn acts to suppress NF‐kβ activity. Our findings build upon this concept, indicating that NF‐kβ is not only a downstream effector of TSPO but also a key mediator of the TSPO–aging interaction in inflammation. Since *NF‐kβ* and interferons have been identified as potential therapeutic targets in AD (Chavoshinezhad et al. 2023; Grimaldi et al. 2014; Mudò et al. 2019; Sun et al. 2022) and play an important role in driving inflammaging and senescence (Salminen et al. 2008, 2012; Songkiatisak et al. 2022) the TSPO–aging interaction in *NF‐kβ* and interferon transcriptional pathways may be an important consideration in therapeutic development.

### 3.7 Drugs That Disrupt Cell Cycle and Induce DNA Damage Mimic TSPO‐Dependent Aging Transcriptional Signature

To further investigate, we used an in silico approach to identify small molecules that phenocopy TSPO‐dependent inflammaging. We compared the TSPO‐dependent inflammaging transcriptome signature with drug gene expression signatures in a perturbational signature library called Connectivity Map (CMap), using the LINCS database (Tham and Langley 2022). The similarity (or dissimilarity) between the TSPO‐KO inflammaging and drug signatures is quantified with a connectivity score, where a score of +1 indicates a strong transcriptional phenocopy, while a score of −1 indicates a strong transcriptional reversal of the TSPO‐KO inflammaging signature. We focused on transcriptional signatures from drugs screened in two key cell types: neural progenitor cells (NPCs) and a glioblastoma cell line (GI1). This approach can help identify drugs that can reverse pathological gene expression patterns and provides insights into the mechanism of action by linking gene expression signatures from the aging TSPO‐KO inflammatory condition with known drug signaling pathways.

**FIGURE 4:**
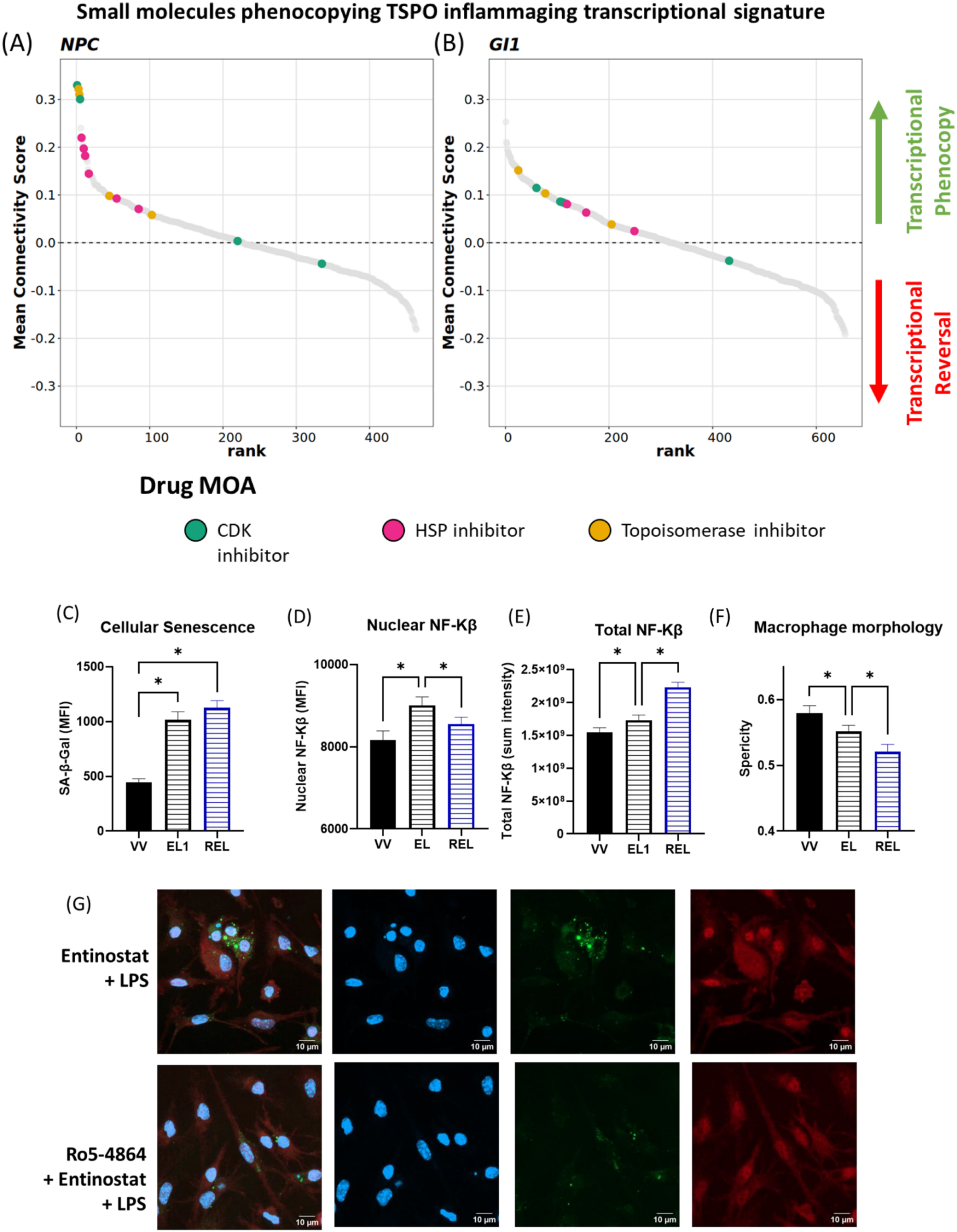
Identification of small molecules that transcriptionally phenocopy TSPO‐KO hippocampal inflammaging to verify TSPO modulation of NF‐κB in senescent microglia. (A) Small molecules identified to transcriptionally phenocopy the TSPO inflammaging signature via Connectivity Mapping. Mean connectivity score against rank of LINCS compounds treated in (A) NPC and (B) GI1 cell line when queried with TSPO inflammaging signature. Positive connectivity score indicates the phenocopying effect of a compound with the query signature, while a negative score indicates the anti‐correlation between the compound and query signature. Compounds are ranked in ascending order, from the strongest phenocopy to strongest reversal signal. Green: CDK inhibitors, Pink: HSP90 inhibitors, Yellow: Topoisomerase inhibitors. (C) Quantification of median intensity of SA‐β‐Gal fluorescence signal (MFI) in cultured WT primary microglia as evaluated by staining of SA‐β‐Gal. Treatment conditions as follows: VV (Vehicle), EL (Entinostat+LPS), REL (Ro5‐4864 + Entinostat+LPS). (D) Quantification of nuclear median intensity of NF‐kβ immunoreactivity (MFI) in primary microglia. (E) Quantification of sum of total per cell NF‐kβ immunoreactivity in primary microglia. (F) Quantification of cellular morphology of the primary microglia as evaluated by whole cell sphericity. (G) Representative confocal images of primary microglia stained for DAPI (blue), NF‐kβ (red) and SA‐β‐Gal (green). Statistical test: Welch's one‐way ANOVA, FDR post hoc multiple comparisons. *p < 0.05.

CDK (cyclin‐dependent kinase) inhibitors, topoisomerase inhibitors, and heat shock protein 90 (HSP90) inhibitors were commonly identified in both cell types analyzed to closely mimic the inflammatory transcriptional signature characterizing TSPO‐dependent aging (Figure 4A,B). These drugs disrupt processes such as cell cycle regulation, DNA integrity, and stress response pathways, and can be used to induce cellular senescence. The highest ranked phenocopy compound identified in the NPCs, Dinaciclib (mean connectivity score = +0.33), is a selective inhibitor of CDK1, CDK2, CDK5, and CKD9. This compound was not tested in the GI1 cell line. CDK inhibitors suppress the activity of key kinases at different stages of the cell cycle process, thus halting cell proliferation (Parry et al. 2010; Webster and Kimball 2000). Another selective CDK1 inhibitor, CGP‐60474, is the 5th ranked phenocopying compound with TSPO‐KO inflammaging (mean connectivity score = +0.30; Figure 4A). Likewise, a number of CDK inhibitors were also identified to significantly phenocopy the TSPO‐dependent inflammaging signature in the glioma GI1 cells, although they were not the strongest phenocopies identified (Table S17). While specific CDK inhibitors were not as strongly correlated with the TSPO‐KO inflammaging signature in the GI1 glioma compared to the NPC, the top ranked phenocopy drug in the GI1 glioma cells is the CHK (Checkpoint) inhibitor, LY‐2606368 (1st, +0.25). Checkpoint kinases are upstream regulators of CDKs (Janetka and Ashwell 2009), corroborating potential disruption of cell cycle processes in TSPO‐KO inflammaging.

Two topoisomerase inhibitors, mitoxantrone and topotecan, also strongly phenocopied the TSPO‐KO inflammaging signature in NPCs (ranked 2nd and 3rd, mean connectivity score = +0.32 and +0.31, respectively; Figure 4A). Although these two topoisomerase inhibitors were not tested in the GI1 glioma cell line, the inhibitors daunorubicin (25th rank, mean connectivity score = +0.15) and camptothecin (77th, +0.10) also significantly phenocopied the TSPO‐dependent inflammaging signature (Figure 4B). Topoisomerase inhibitors are DNA damage–inducing compounds that act by trapping the Top1‐DNA cleavage complex (Top1cc) and preventing the cleaved DNA from re‐ligating, typically used to trigger cancer cell apoptosis (Katyal et al. 2014). This potentially indicated elevated DNA damage in the TSPO‐KO inflammaging hippocampus.

Interestingly, two heat shock protein 90 (HSP90) inhibitors were also highly ranked, phenocopying the TSPO‐KO inflammaging signature—tanespimycin (ranked 7th, mean connectivity score = +0.22) and geldanamycin (ranked 10th, mean connectivity score = +0.20; Figure 3A). These compounds bind to the HSP90, which in turn releases the HSF1 (heat shock factor 1) transcription factor to activate the expression of other HSPs during cellular stress (Kurop et al. 2021). This finding suggests that cellular stress triggering the heat shock response may also be associated with the loss of TSPO function during inflammaging.

Overall, the classes of drugs found to mimic TSPO‐dependent aging induced key hallmarks of cellular senescence, including cell cycle disruption and DNA damage. Based on this, we hypothesized that TSPO deletion in aging promotes innate immune cell senescence via NF‐Kβ signaling, which we had identified as a hub in the TSPO–aging transcriptional networks. In subsequent studies, we test this hypothesis in cultured microglia and macrophages.

### 3.8 TSPO Modulates NF‐kβ Activation in Microglia and Macrophages

To test the hypothesis that TSPO deletion promotes innate immune cell senescence in aging via NF‐Kβ signaling, we investigated the effect of Entinostat, a histone deacetylase (HDAC) inhibitor, which was identified in our analysis to strongly phenocopy the TSPO‐KO aging transcriptomic signature. Entinostat was selected for further investigation because it is known to affect NF‐Kβ signaling pathways, innate immune activation, and cellular senescence (Benjaskulluecha et al. 2022; Bhat et al. 2024; Dai et al. 2005; Min et al. 2021; Ryu et al. 2019; Sidiropoulos et al. 2022; Stanfield et al. 2021). Of particular interest, a previous study has demonstrated that entinostat enhances LPS‐induced inflammatory responses in macrophages (Benjaskulluecha et al. 2022).

First, we established an in vitro model of aged microglia by treating primary microglia with entinostat combined with LPS to induce cellular senescence. As a biomarker of cellular senescence, we measured β‐galactosidase activity (SA‐β‐Gal), a hallmark of aging and senescent cells. Entinostat combined with LPS increased SA‐β‐Gal activity, which was not reversed by the TSPO agonist, Ro5‐4864 (one‐way ANOVA, *F* = 35.42, *p* < 0.0001, FDR posthoc, vehicle vs. entinostat + LPS or vehicle vs. entinostat + LPS + Ro5‐4864: *p* < 0.0001; *n* = 96–135 cells/group; Figure 4E). Notably, although the TSPO agonist did not alter total SA‐β‐Gal activity, it resulted in smaller, yet more abundant SA‐β‐Gal‐positive lysosomes.

Next, we tested the effect of Ro5‐4864 on the nuclear localization of the NF‐kβ subunit P65 in senescent microglia treated with ENTINOSTAT + LPS. Increased nuclear NF‐kβ was observed in entinostat + LPS‐treated microglia, which was reversed by Ro5‐4864 (one‐way ANOVA, *F* = 4.86, *p* = 0.008, FDR post hoc, vehicle vs. entinostat + LPS: *p* = 0.006; entinostat + LPS vs. entinostat + LPS + Ro5‐4864: *p* = 0.04; *n* = 96–135 cells/group; Figure 4C,F). Increased nuclear NF‐kβ in entinostat + LPS‐treated microglia was coupled with an increase in total cellular NF‐kβ, while following Ro5‐4864 treatment, reduced nuclear NF‐kβ was observed despite further elevations in total cellular NF‐kβ (one‐way ANOVA, *F* = 20.25, *p* < 0.0001, FDR post hoc, vehicle vs. entinostat + LPS: *p* = 0.04; entinostat + LPS vs. entinostat + LPS + Ro5‐4864: *n* = 96–135 cells/group; *p* < 0.0001; Figure 4C,F).

This was associated with morphological changes, as Ro5‐4864 treated microglia appeared rod‐like with fewer processes, which was reflected in significantly increased circularity in Ro5‐4864 treated microglia (one‐way ANOVA, *F* = 7.06, *p* = 0.001, FDR post hoc, entinostat + LPS vs. entinostat + LPS + Ro5‐4864: *p* = 0.04; *n* = 96–135 cells/group; Figure 4D,F). These findings support a role for TSPO in modulating NF‐kβ signaling in senescent microglia.

**FIGURE 5:**
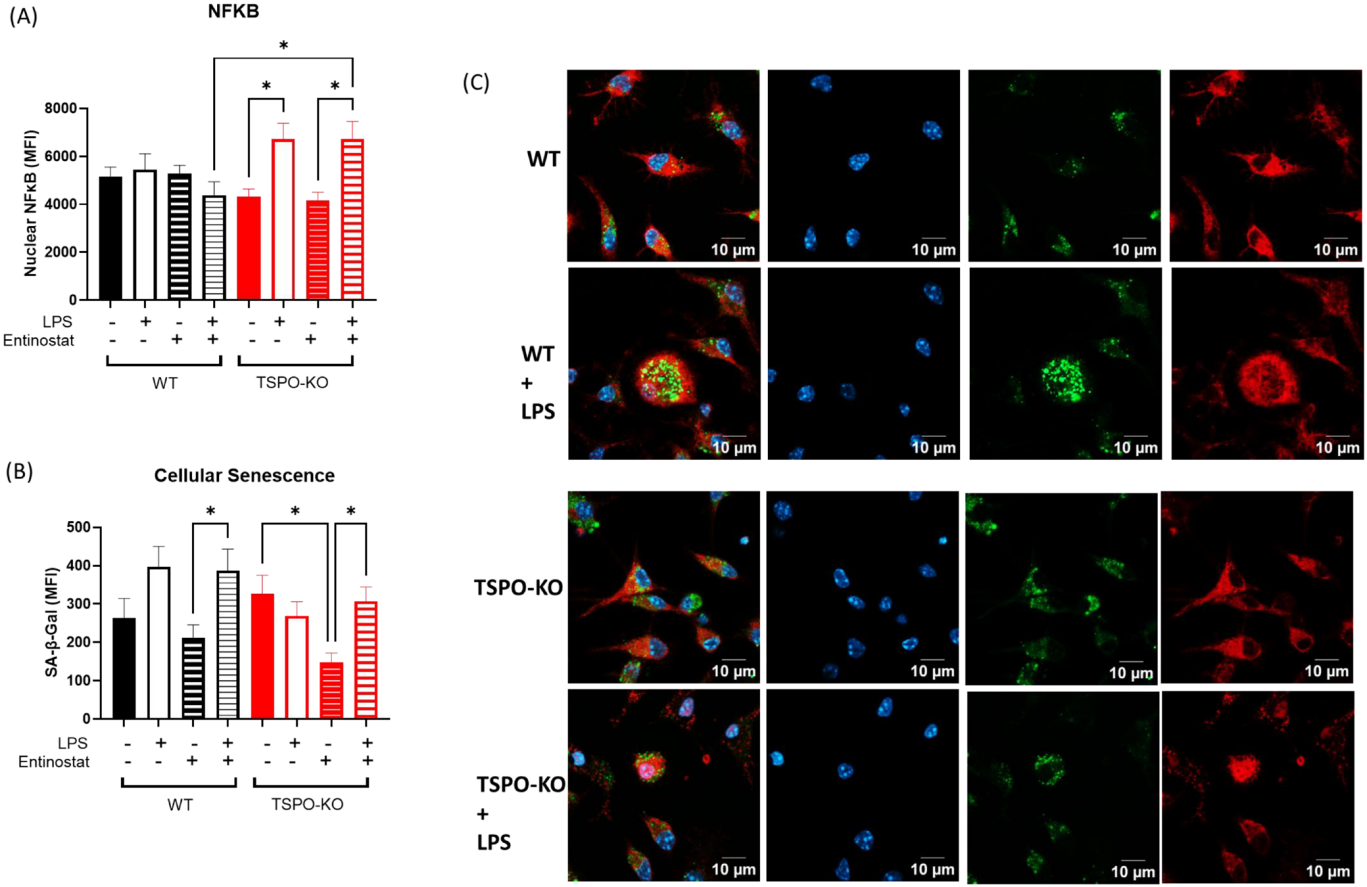
Effect of TSPO deletion on nuclear NF‐kβ activation and cellular senescence in cultured aged macrophages. Immunocytochemistry and staining of SA‐β‐Gal were performed on cultured macrophages derived from aged WT and sTSPO‐KO mice. (A) Quantification of nuclear median intensity of NF‐kβ immunoreactivity (MFI). (B) Quantification of cellular senescence measured by SA‐β‐Gal fluorescence (median fluorescence intensity, MFI). (C) Representative confocal images of the cultured macrophages derived from aged WT and TSPO‐KO mice. DAPI (blue), NF‐kβ (red) and SA‐β‐Gal (green). Statistics: Welch's one‐way ANOVA, FDR post hoc multiple comparisons *p < 0.05.

To genetically investigate the potential role of TSPO in NF‐kβ signaling, we compared nuclear NF‐kβ activation in primary macrophages derived from aged WT and TSPO‐KO mice. We found that LPS (alone or in combination with Entinostat) robustly increased nuclear NF‐kβ in TSPO‐KO but not WT macrophages (one‐way ANOVA, *F* = 3.92, *p =* 0.0004, FDR post hoc: WT vehicle vs. LPS *p* = 0.70, TSPO‐KO vehicle vs. LPS *p* = 0.02; *n* = 55–119 cells/group; Figure 5A,D). Total cellular NF‐kβ levels were not significantly changed between treatment conditions (one‐way ANOVA, F = 1.88, *p* = 0.07; *n* = 55–119 cells/group; Figure S4A,C). Macrophages treated with LPS and Entinostat, alone or in combination, appeared more ameboid; this was measurable in TSPO‐KO macrophages treated with Entinostat + LPS, which had significantly increased sphericity compared to vehicle‐treated WT macrophages (one‐way ANOVA, *F* = 2.31, *p =* 0.03; FDR post hoc: WT vehicle vs. TSPO‐KO entinostat + LPS *p* = 0.03; *n* = 55–119 cells/group; Figure S4B). Surprisingly, Entinostat reduced the senescence marker, SA‐β‐Gal, in TSPO‐KO but not WT macrophages (one‐way ANOVA, F = 3.75, *p =* 0.0006; FDR post hoc: WT vehicle vs. WT Entinostat: *p* = 0.39, TSPO‐KO vehicle vs. TSPO‐KO Entinostat: *p* = 0.009; *n* = 55–119 cells/group; Figure 4B,C). This corresponded to reduced heterochromatin foci in entinostat‐treated TSPO‐KO but not WT macrophages, corroborating reduced senescence and suggesting that entinostat induced increased chromatin relaxation in TSPO‐KO compared to WT macrophages (one‐way ANOVA, *F* = 4.51, *p =* 0.01; FDR post hoc: WT vehicle vs. WT entinostat: *p* = 0.13, TSPO‐KO vehicle vs. TSPO‐KO entinostat: *p* = 0.02; *n* = 8–12 images from 3 replicates/group; Figure S4C). LPS treatment increased senescence measured by SA‐β‐Gal activity in both WT and TSPO‐KO macrophages treated with entinostat.

### 3.9 Aging and TSPO Alter Brain Metabolic Profiles in Inflammation

**FIGURE 6:**
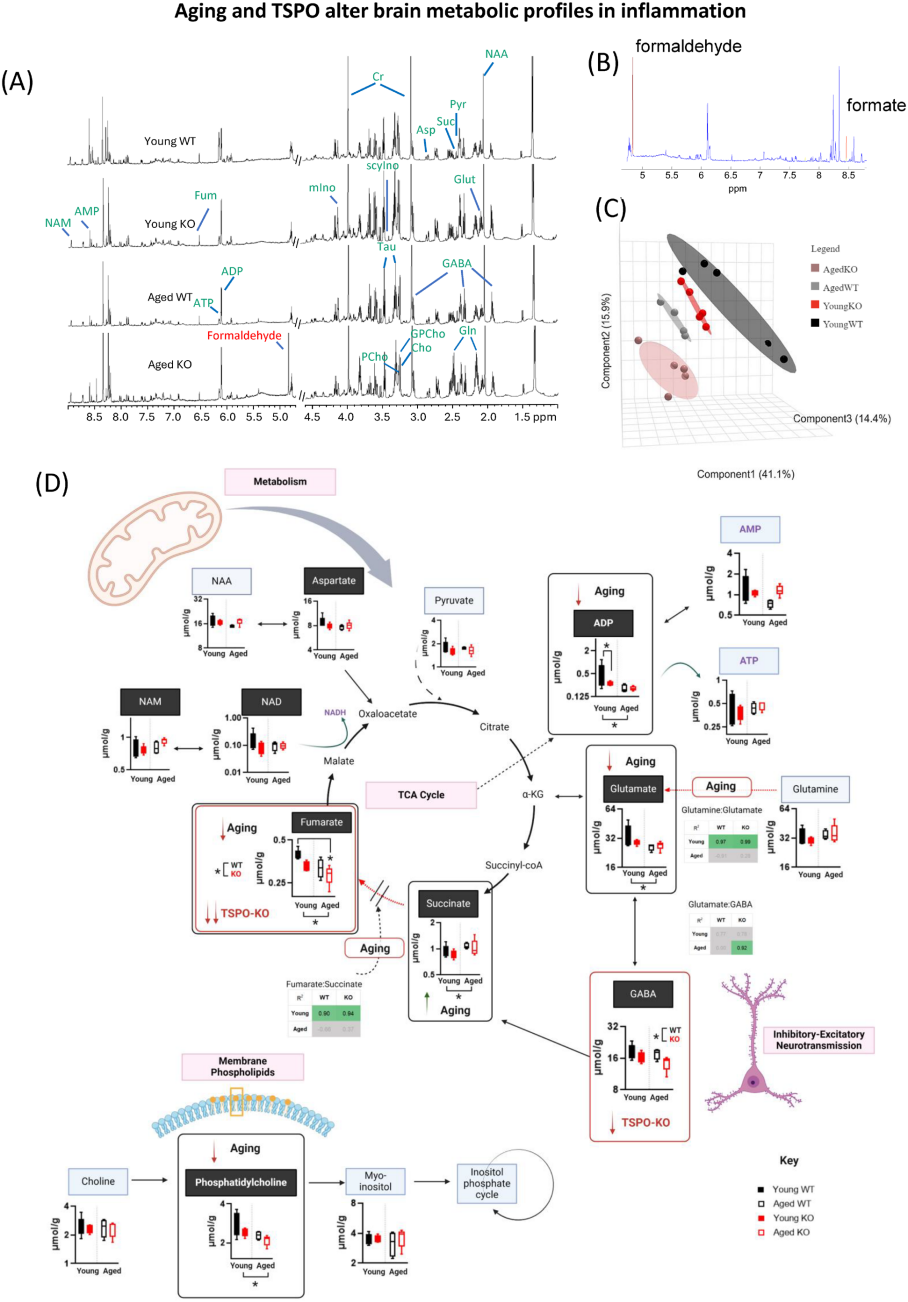
Aging and TSPO alter brain metabolic profiles in inflammation. (A) Representative 1D‐NOSEY spectra of the 19 metabolites detected in young WT, old WT, young KO and old KO brain tissue samples. Metabolite assignment to NMR peaks indicated. ADP, Adenosine diphosphate; AMP, Adenosine monophosphate; ATP, Adenosine triphosphate; Cho, choline; Cr, creatine; Fum, fumarate; GABA, γ‐aminobutyrate; gln, glutamine; glu, glutamate; GPCho, glycerophosphocholine; mIno, myo‐inositol; NAA, N‐acetyl aspartate; Nam, nicotinamide; PCho, phosphocholine; Pyr, pyruvate; scyIno, scyllo‐inositol; Suc, succinate; Tau, taurine. (B) STOCSY shows high correlation (0.8) between the two singlets (red peaks) at 4.832 (identified in aged TSPO‐KO only) and 8.462 ppm, which is formic acid. The correlation suggests that peak at 4.832 could be formaldehyde. Formaldehyde in water presents structure of methanediol and it is a singlet with chemical shift ranging from 4.4–5.4 ppm (Automated Topology Builder (ATB) and Repository Version 3.0, Methanediol | CH4O2 | MD Topology | NMR | X‐Ray (uq.edu.au)). (C) Multivariate dimensionality reduction of targeted NMR metabolites using partial least squares‐discriminant analysis (PLSDA) for young and aged WT and TSPO‐KO mice under LPS‐induced inflammation. (D) Quantification of metabolites measured by NMR in young and aged brain of WT and TSPO‐KO mice under LPS‐induced inflammation. Metabolite concentrations shown as median, interquartile range with error bars indicating minimum and maximum. Metabolites that contributed the greatest variance to discrimination of the groups along PC1 are highlighted with black header (aspartate, NAM, NAD, ADP, glutamate, GABA, succinate, fumarate and phosphatidylcholine). Correlation heatmaps show precursor:product ratios as measured by Pearson's correlation, with significance threshold *p* < 0.05. Precursor:product metabolite ratios which showed significant changes during aging were fumarate:succinate, and glutamine:glutamate (fumarate:succinate and glutamine:glutamate significantly correlated in the young but not aged). Conversely, glutamate:GABA ratio showed significant correlation exclusively in the TSPO aged group. Created in BioRender. https://BioRender.com/q60q212.

Since our transcriptomic data identified TSPO‐dependent changes in metabolic pathways in the aged brain under inflammation, NMR was used to determine brain metabolites involved in the metabolism of carbohydrates, amino acids, and lipids in young and aged WT and TSPO‐KO mice treated with LPS. We identified 20 metabolites, which include amino acids (glutamine, glutamate, aspartate, N‐acetylaspartate (NAA), gamma‐aminobutyric acid (GABA), tricarboxylic acid cycle (TCA) metabolites (fumarate, succinate), redox cofactors (NAD), nicotinamide (NAM), adenosine diphosphate (ADP), and membrane components (choline, glycerophosphatidylcholine, phosphatidylcholine) (Figure 6A). Additionally, a peak corresponding to formaldehyde, which is usually undetectable in the brain, was identified exclusively in the aged TSPO‐KO mice (Figure 6B).

PLS‐DA was used to determine the major metabolites discriminating between young versus aged and WT versus TSPO‐KO conditions (Figure 6C). Each group clustered with distinct separation by genotype for each age. Aging was most clearly discriminated along the principal component axis 1 and 2, which explained 41.1% and 15.9% of the variability, respectively (Figure 6C). Metabolites that contributed the greatest variance to discrimination of the groups along PC1 were involved in the TCA cycle (Fumarate, Succinate, Glutamate, GABA, Aspartate), membrane phospholipids (Phosphatidylcholine, Glycerolphosphocholine), and redox cofactors (NAD, NAM, ADP; Variance Importance Projections (VIP) score > 0.8; Figure 6A).

Comparison of metabolite concentrations in aging revealed increased levels of the TCA metabolite, succinate, coupled with reduced levels of its product, fumarate, suggesting inefficient conversion of succinate to fumarate in the aged inflammatory brain (PERMANOVA: main effect: aging; Fumarate, *F*_(1,5.66)_ = 8.41, *p* = 0.007; succinate, *F*_(1,5.66)_ = 4.98, *p* = 0.043; Figure 6D; Table S18). Supporting this, succinate and fumarate levels were significantly correlated in young but not aged brains in both WT and TSPO‐KO mice, suggesting a break in the TCA cycle at succinate dehydrogenase in aged mice (Figure 6D). TSPO deletion was also associated with depleted levels of fumarate (PERMANOVA: main effect: genotype; Fumarate, *F*_(1,5.66)_ = 5.82, *p* = 0.026; Figure 6D, Table S18). Further, age‐related depletion of fumarate was more severe in TSPO‐KO brains, with reduced levels detected in young and aged TSPO‐KO mice compared to young WT (PERMANOVA: age × genotype interaction; Table S18; fumarate, *F*_(3,5.66)_ = 6.70, *p* = 0.003; young WT vs. aged TSPO‐KO, FDR = 0.0067). Interestingly, fumarate is an anti‐inflammatory metabolite, shown to inhibit activation of NF‐κβ and approved for the treatment of multiple sclerosis (Miljković et al. 2015). Therefore, depleted levels of this metabolite could potentially contribute to the increased inflammatory transcriptional signatures detected in TSPO‐KO inflammaging.

Age‐related reductions in levels of glutamate, which fuels the TCA cycle via α‐ketoglutarate, in addition to being the major excitatory neurotransmitter of the brain, were also observed (PERMANOVA: main effect: aging; glutamate, *F*_(1,5.66)_ = 5.70, *p* = 0.011; Figure 6D; Table S18). Glutamate concentrations significantly correlated with levels of its precursor, glutamine, in young but not aged brains of both WT and TSPO‐KO mice, potentially indicating reduced efficiency of glutamate synthesis in the aging brain (Figure 6D). In contrast, TSPO deletion resulted in significantly reduced levels of the glutamate metabolite, GABA, which feeds into the TCA cycle via succinate, in addition to being the major inhibitory neurotransmitter of the brain (PERMANOVA: main effect: genotype; GABA, *F*_(1,5.66)_ = 6.02, *p* = 0.015; Figure 6D; Table S18). Depleted brain GABA levels in TSPO‐KO mice were consistent with our transcriptomic findings, that identified a downregulation of pathways related to the GABAergic synapse in the hippocampus of aged TSPO‐KO versus WT mice. These changes in glutamate and GABA levels could potentially reflect an excitatory‐inhibitory imbalance, which is associated with anxiety and depression (Zhang, Liu, et al. 2021). We have previously shown that TSPO deletion increases anxiety‐related behavior in mice (Barron et al. 2021); thus, changes in the bioavailability of glutamate and GABA may contribute to this effect.

Reduced accumulation of the energy substrate, ADP, was also observed in the aging brain (PERMANOVA: main effect: aging; ADP, *F*_(1,5.66)_ = 4.65, *p* = 0.005; Figure 6D). ADP levels significantly correlated with concentrations of its metabolite, AMP, but not ATP, in the brains of young WT mice only. A significant interaction between TSPO‐KO and aging was identified for ADP, with pairwise analysis indicating ADP was significantly reduced due to aging in WT mice only (PERMANOVA: age × genotype interaction; ADP, *F*_(1,5.66)_ = 2.60, *p* = 0.026; Figure 6D).

Lastly, reduced levels of the membrane phospholipid, phosphatidylcholine, were also observed in the aging brain in inflammation (PERMANOVA: main effect: aging; phosphatidylcholine), *F*_(1,5.66)_ = 6.77, *p* = 0.013; (Figure 6D). In young mice but not aged mice, phosphatidylcholine concentrations positively correlated with glycerophosphatidylcholine concentrations (WT young *r* ^2^ = 0.89, *p* = 0.04; TSPO‐KO young *r* ^2^ = 0.93, *p* = 0.02; WT old *r* ^2^ = −0.79, *p* = 0.20; TSPO‐KO young *r* ^2^ = 0.056, *p* = 0.92). A significant interaction between TSPO‐KO and aging was also identified for phosphatidylcholine (PERMANOVA: age × genotype interaction; phosphatidylcholine), *F*_(1,5.66)_ = 3.63, *p* = 0.035 (Figure 6D). Previous studies have demonstrated that hippocampal phospholipid levels, including phosphatidylcholine, decline in aging. In addition to comprising the cell membrane, phosphatidylcholine is also a precursor for the major neurotransmitter acetylcholine (Chung et al. 1995), which is important in memory and depleted in AD (Giacobini et al. 2022; Liu et al. 2022). Interestingly, phosphatidylcholine has been shown to inhibit LPS‐induced inflammation and improve cognitive function (Tan et al. 2020), opening the possibility that age‐related decline in hippocampal phosphatidylcholine may also play a role in inflammaging.

## 4 Conclusions

Broadly, our study indicates TSPO plays a protective role in brain aging and that TSPO deletion exacerbates age‐related transcriptional and metabolic changes in the inflamed hippocampus. This is of significance since we found TSPO levels in brain innate immune cells declined in aging. TSPO deletion aggravated key age‐related synaptic and immune transcriptional signatures in the inflamed hippocampus, and these inflammaging signatures were mimicked by drugs that disrupt the cell cycle, cause DNA damage, and cell stress through the heat shock response. TSPO deletion also worsened age‐related changes in brain metabolites, including depletion of the major inhibitory neurotransmitter GABA and the anti‐inflammatory TCA cycle metabolite fumarate, providing a potential link between TSPO mitochondrial function and immune regulation. Importantly, we found an interaction between TSPO function and aging in the hippocampus. Aging resulted in a reversal of TSPO‐KO transcriptional signatures following inflammatory insult. While we have previously shown that TSPO deletion dampens hippocampal inflammatory signatures in the young adult, we were surprised to discover that loss of TSPO drastically exacerbated inflammatory transcriptional responses in the aging hippocampus. This TSPO–aging interaction was linked to NF‐kβ and interferon regulatory transcriptional networks. We verified TSPO‐dependent modulation of NF‐kβ activation in cultured microglia and macrophages. NF‐kβ and interferons have been implicated in brain aging as well as the pathogenesis of AD. This TSPO–aging interaction is an important consideration in the interpretation of TSPO‐targeted biomarker and therapeutic studies, as well as in vitro studies that cannot easily model brain aging.

## Author Contributions

**Kei Onn Lai:** conceptualization, Methodology, Investigation, Validation, Formal Analysis, Visualization, Writing – original draft. **Jia Hui Wong:** methodology, Investigation, Validation, Formal Analysis, Visualization, Writing – revised draft. **Nevin Tham:** methodology, Investigation, Validation, Formal Analysis, Visualization, Writing – original draft. **Lauren Fairley:** methodology, Investigation; **Roshan Ratnakar Naik:** methodology, Investigation. **Yulan Wang:** methodology, Investigation, Validation, Formal Analysis, Visualization, Writing – review and editing. **Sarah R. Langley:** methodology, Writing – review and editing, Supervision. **Anna M. Barron:** conceptualization, Formal Analysis, Visualization, Writing – original draft, Supervision, Funding acquisition.

## Conflicts of Interest

The authors declare no conflicts of interest.

## Supporting information


**Table S1.** Differentially expressed gene list for normal aging. Comparison is between WT Aged versus WT Young (reference) under baseline (PBS) conditions. Significance is defined as FDR < 0.05. Upregulation refers to significant genes thresholded at Log2FC ≥ 0.5, while downregulation refers to significant genes thresholded at Log2FC ≤ −0.5.
**Table S2.** fGSEA functional enrichment results from differentially expressed genes of normal aging comparison (Table S1). NES score refers to normalized enrichment score. Leading edge refers to genes annotated with their Entrez IDs being involved in that functional term.
**Table S3.** fGSEA leading edges with gene symbols from Table S2. NES score refers to normalized enrichment score. MGI refers to the overlapping genes involved in the functional term.
**Table S4.** Gene overlap hypergeometric Fischer tests of significant differentially expressed genes between normal aging and inflammatory response in the young. WT PBS Aged versus WT PBS Young is denoted as the normal aging comparison, while WT LPS Young versus WT PBS Young is denoted as the inflammatory response in the young comparison. Each significant differentially expressed gene set is split into either upregulated “up” (Log2FC > 0.5) or downregulated “down” (Log2FC < 0.5). Magnitude of overlap is reflected as odds ratio, while significance threshold is *p* < 0.05 for the hypergeometric Fischer test.
**Table S5.** fGSEA functional enrichment results from differentially expressed genes of inflammatory response in the young. NES score refers to normalized enrichment score. Leading edge refers to genes annotated with their Entrez IDs being involved in that functional term.
**Table S6.** Differentially expressed gene list for inflammation in the aged brain. Comparison is between WT LPS Aged versus WT PBS Aged conditions. Significance is defined as FDR < 0.05. Upregulation refers to significant genes thresholded at Log2FC ≥ 0.5, while downregulation refers to significant genes thresholded at Log2FC ≤ −0.5.
**Table S7.** Differentially expressed gene list for aging in the inflamed hippocampi. Comparison is between WT LPS Aged versus WT LPS Young conditions. Significance is defined as FDR < 0.05. Upregulation refers to significant genes thresholded at Log2FC ≥ 0.5, while downregulation refers to significant genes thresholded at Log2FC ≤ −0.5.
**Table S8.** Differentially expressed gene list for the effect of TSPO deletion in the aged brain. Comparison is between KO PBS Aged versus WT PBS Aged conditions. Significance is defined as FDR < 0.05. Upregulation refers to significant genes thresholded at Log2FC ≥ 0.5, while downregulation refers to significant genes thresholded at Log2FC ≤ −0.5.
**Table S9.** Differentially expressed gene list for the effect of TSPO deletion in the inflamed aged brain. Significant (FDR < 0.05) differentially expressed genes TSPOKO LPS Aged versus WT LPS Aged. Upregulation refers to significant genes thresholded at Log2FC ≥ 0.5, while downregulation refers to significant genes thresholded at Log2FC ≤ −0.5.
**Table S10.** fGSEA functional enrichment results from differentially expressed genes evaluating the effect of TSPO deletion in the aged brain. Gene input is based on all differentially expressed genes comparing TSPOKO Aged versus WT Aged. NES score refers to normalized enrichment score. MGI refers to the overlapping genes involved in the functional term.
**Table S11.** Gene overlap hypergeometric Fischer tests comparing the effect of TSPO deletion across aging of the inflamed brain. Gene overlap is performed to compare overlapping differentially expressed genes between TSPOKO LPS Aged versus WT LPS Aged comparison and TSPOKO LPS Young versus WT LPS Young comparison. Each significant differentially expressed gene set is split into either upregulated “up” (Log2FC > 0.5) or downregulated “down” (Log2FC < 0.5). Magnitude of overlap is reflected as odds ratio, while significance threshold is *p* < 0.05 for the hypergeometric Fischer test.
**Table S12.** Functional enrichment of the markers belonging to the subcategories of cell types. Subcategories of cell types detected via deconvolution of our bulk RNAseq dataset expression matrix using (Zeisel et al. 2015). Markers are derived using Seurat by comparing each subcategory of cell type against all other subcategories using “FindMarkers” function.
**Table S13.** Module Eigengenes calculated for each module. Module Eigengenes (PC1) calculated for each module, based on expression. Includes sample metadata for mapping sample groups, and the pairwise marginal means (emmeans) for post hoc analysis after PERMANOVA.
**Table S14.** Over representation Gene Ontology functional enrichment of significant modules (Modules 1–6). Gene Ontology functional enrichment using C5 of MsigDb. Overlap genes shows module genes involved in the functional term. Overlap represents size of overlap genes while size represents size of the gene set. Significance is determined by FDR *p* adjusted value (*p*adj < 0.05).
**Table S15.** Intra‐module connectivity of module members. All module members, and the respective size of each module is documented. Next, members with high intra‐module connectivity (where gene members > 0.9 correlation with PC1 of module, were filtered out).
**Table S16.** Transcription factors enriched are predicted from Module 5 via HOMER Motif analysis. Transcription factors that are significantly enriched from Module 5 members’ promoters (Benjamini Hochberg *p* adjusted value < 0.05) are selected. Cognate genes from these transcription factors are isolated from the expression matrix, and integrated with Module 5 members as a Network. Network are derived via Network Analyst website, by querying through STRING database for PPI. Network topology is documented with details of describing Hub genes measurement such as degree (number of PPI interactions) and Betweenness Centrality (measure of centrality of gene within network).
**Table S17.** Connectivity Map query of the top 100 differentially expressed genes in TSPOKO inflammaging. Querying is conducted via (Zhang and Gant 2008) method in either Neural precursor cell line (NPC) or Glioblastoma cell line (Gli1). Maximal phenocopy or reversal of transcriptional signature is +1 or −1 of the mean connectivity score respectively. Smaller rank magnitude represents a transcriptional phenocopy while larger rank magnitude represents a transcriptional reversal.
**Table S18.** Data of targeted NMR of 20 metabolites. NMR of 20 metabolites are as described in Figure 5. Raw data, shifted log (*x* + 1) transformed data for plotting, and results of univariate permutational ANOVA and post hoc Dunn test are provided.

**Figure S1.** Cell‐type contributions to hippocampal aging transcriptomic signatures in WT & TSPO‐KO mice. Estimation of hippocampal cell‐specific contributions to aging transcriptional signatures in WT and TSPO‐KO mice under baseline conditions. Units of the graphs are arbitrary. Data shown as median, interquartile range with error bars indicating minimum and maximum. Statistical tests: Two‐way ANOVA. *p < 0.05.

**Figure S2.** PPI interaction subnetwork of immune (*top*) and transcriptional‐translational processes (*bottom*) derived from the module driver genes of Module 3 identified by multiWGCNA.

**Figure S3.** PPI subnetwork of immune‐related transcription factors and helicases in Module 4 genes identified by multiWGCNA.

**Figure S4.** Effect of TSPO deletion on nuclear NF‐kβ activation and cellular senescence in cultured aged macrophages. (A) uantification of sum of per cell NF‐kβ immunoreactivity in primary cultured macrophages. (B) Quantification of cellular morphology of the cultured macrophages as evaluated by whole cell sphericity. Values closer to 1.0 have higher sphericity while lower values represent ellipticity. (C) Quantification of senescence associated heterochromatic foci (SAHF) in cultured macrophages measured by punctate DAPI staining.

## Data Availability

The study data will be made available via the https://researchdata.ntu.edu.sg/dataverse/neurobiologyaging repository.
