# Supplementary figures and images for: Age‐Dependent Regulation of Hippocampal Inflammation by the Mitochondrial Translocator Protein in Mice
Source: Aging Cell. 2025 Apr 24;24(6):e70039. doi: 10.1111/acel.70039 (PMC12151901; doi:10.1111/acel.70039)

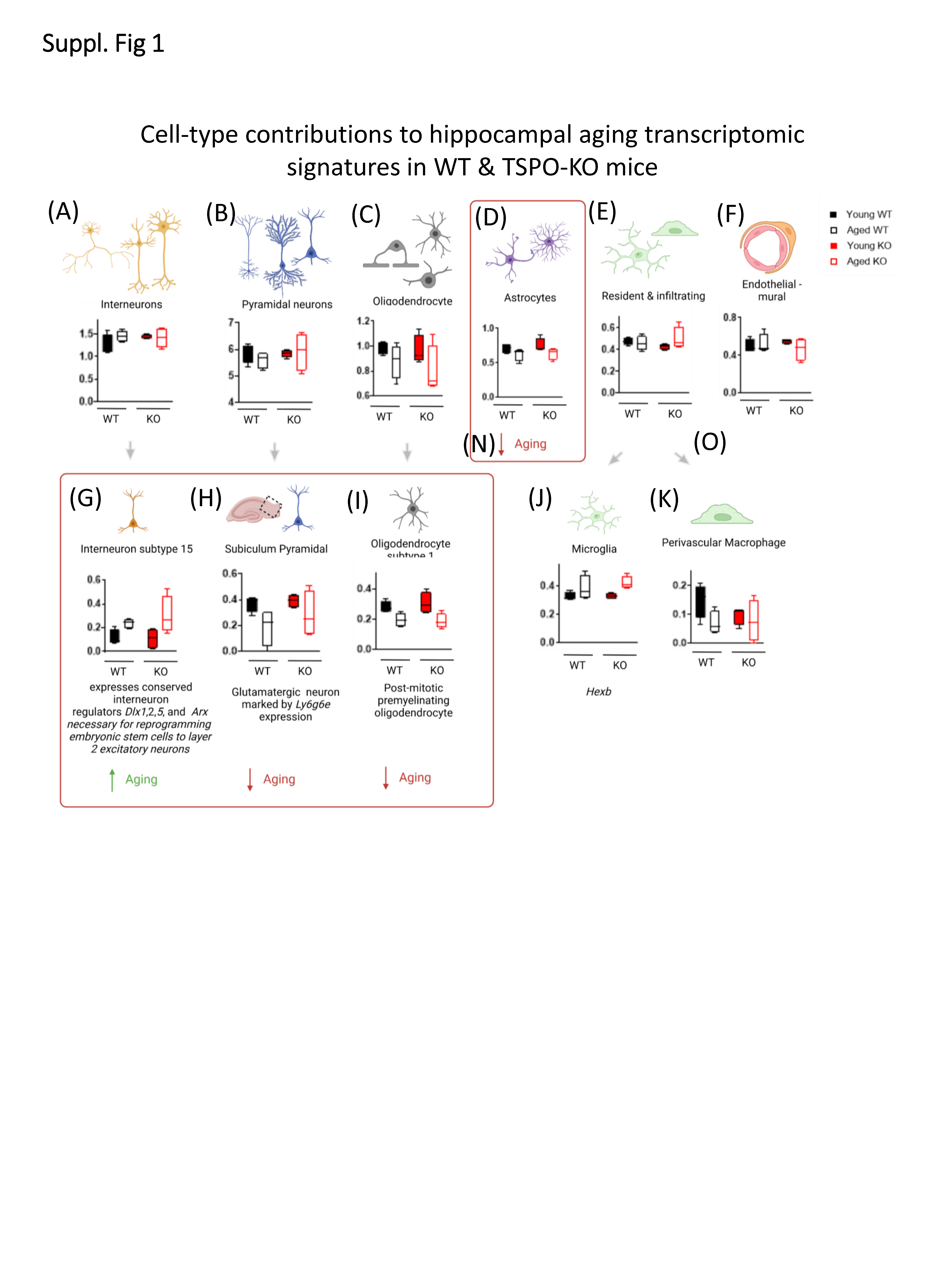

Supplement: Supplementary file 2 — Figure S1. Cell‐type contributions to hippocampal aging transcriptomic signatures in WT & TSPO‐KO mice. Estimation of hippocampal cell‐specific contributions to aging transcriptional signatures in WT and TSPO‐KO mice under baseline conditions. Units of the graphs are arbitrary. Data shown as median, interquartile range with error bars indicating minimum and maximum. Statistical tests: Two‐way ANOVA. *p < 0.05. [file ACEL-24-e70039-s002.tif]

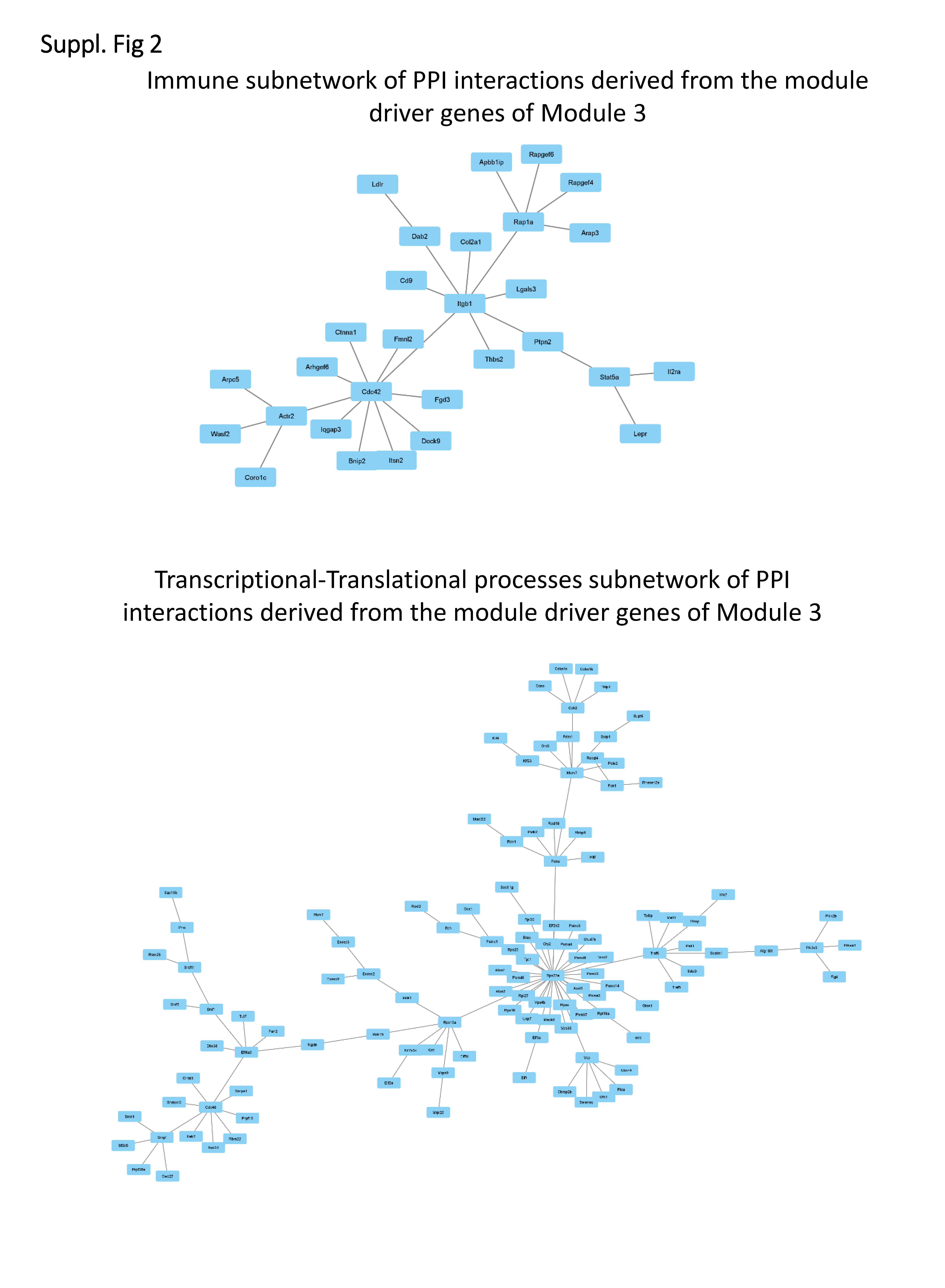

Supplement: Supplementary file 3 — Figure S2. PPI interaction subnetwork of immune (top) and transcriptional‐translational processes (bottom) derived from the module driver genes of Module 3 identified by multiWGCNA. [file ACEL-24-e70039-s005.tif]

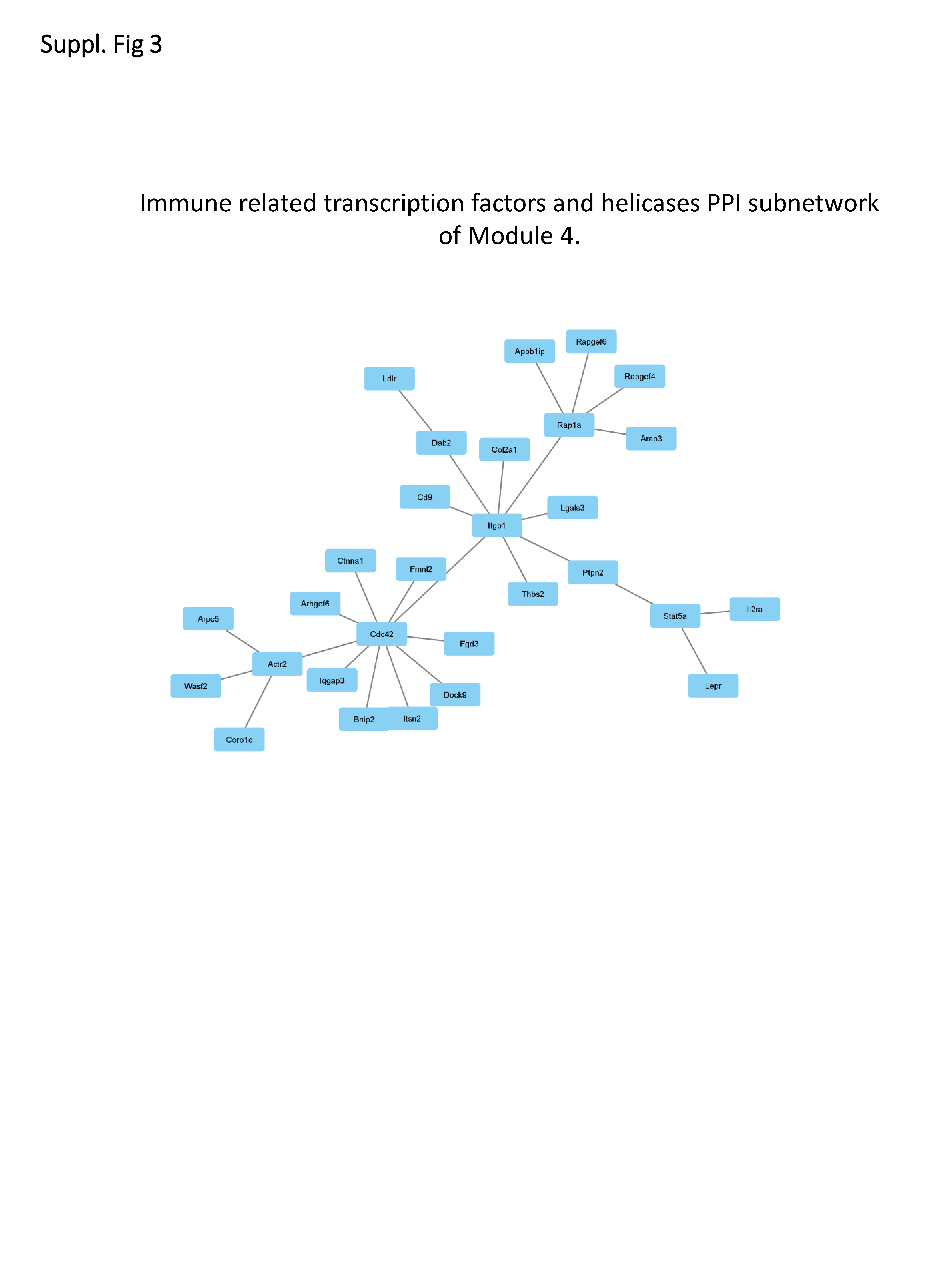

Supplement: Supplementary file 4 — Figure S3. PPI subnetwork of immune‐related transcription factors and helicases in Module 4 genes identified by multiWGCNA. [file ACEL-24-e70039-s004.tif]

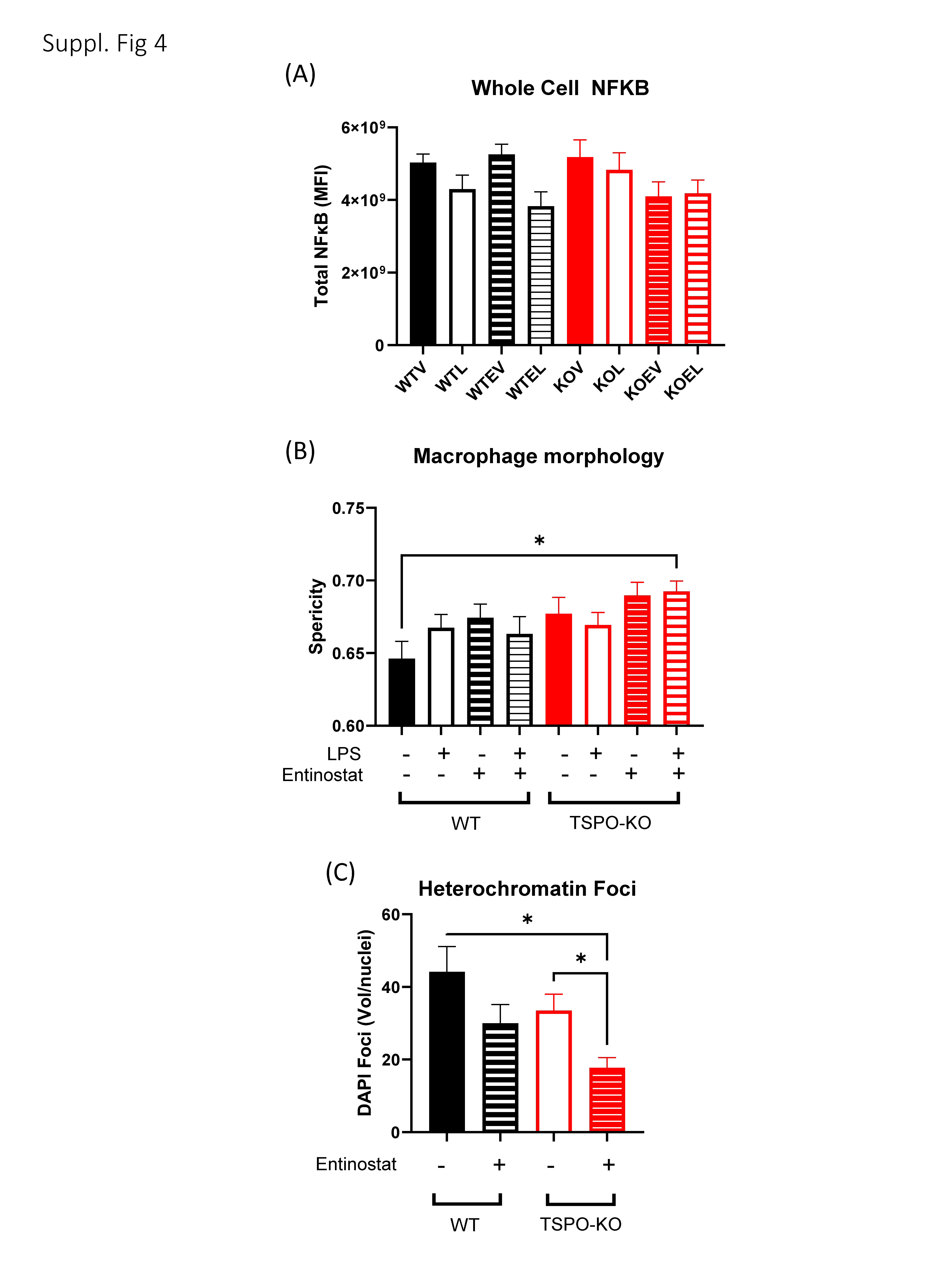

Supplement: Supplementary file 5 — Figure S4. Effect of TSPO deletion on nuclear NF‐kβ activation and cellular senescence in cultured aged macrophages. (A) uantification of sum of per cell NF‐kβ immunoreactivity in primary cultured macrophages. (B) Quantification of cellular morphology of the cultured macrophages as evaluated by whole cell sphericity. Values closer to 1.0 have higher sphericity while lower values represent ellipticity. (C) Quantification of senescence associated heterochromatic foci (SAHF) in cultured macrophages measured by punctate DAPI staining. [file ACEL-24-e70039-s001.tif]
